# MYH1G-AS is a chromatin-associated lncRNA that regulates skeletal muscle development in chicken

**DOI:** 10.1186/s11658-023-00525-x

**Published:** 2024-01-04

**Authors:** Bolin Cai, Manting Ma, Rongshuai Yuan, Zhen Zhou, Jing Zhang, Shaofen Kong, Duo Lin, Ling Lian, Juan Li, Xiquan Zhang, Qinghua Nie

**Affiliations:** 1 State Key Laboratory of Livestock and Poultry Breeding, Guangdong Laboratory for Lingnan Modern Agriculture, College of Animal Science, South China Agricultural University, Guangzhou, China; 2 Guangdong Provincial Key Lab of Agro-Animal Genomics and Molecular Breeding, Key Laboratory of Chicken Genetics, Breeding and Reproduction, Ministry of Agriculture and Rural Affairs, National-Local Joint Engineering Research Center for Livestock Breeding, Guangzhou, China; 3 Randall Centre of Cell and Molecular Biophysics, Faculty of Life Sciences and Medicine, New Hunt’s House, King’s College London, Guy’s Campus, London, UK; 4 National Engineering Laboratory for Animal Breeding and MOA Key Laboratory of Animal Genetics and Breeding, College of Animal Science and Technology, China Agricultural University, Beijing, China; 5 Key Laboratory of Bio-Resources and Eco-Environment of Ministry of Education, College of Life Sciences, Sichuan University, Chengdu, China

**Keywords:** Chromatin accessibility, LncRNA MYH1G-AS, m^6^A methylation, Skeletal muscle development

## Abstract

**Background:**

Skeletal muscle development is pivotal for animal growth and health. Recently, long noncoding RNAs (lncRNAs) were found to interact with chromatin through diverse roles. However, little is known about how lncRNAs act as chromatin-associated RNAs to regulate skeletal muscle development. Here, we aim to investigate the regulation of chromatin-associated RNA (MYH1G-AS) during skeletal muscle development.

**Methods:**

We provided comprehensive insight into the RNA profile and chromatin accessibility of different myofibers, combining RNA sequencing (RNA-seq) with an assay for transposase-accessible chromatin with high-throughput sequencing (ATAC-seq). The dual-luciferase reporter assay and chromatin immunoprecipitation (ChIP) assay were used to analyze the transcriptional regulation mechanism of MYH1G-AS. *ALKBH5*-mediated MYH1G-AS *N*^6^-methyladenosine (m^6^A) demethylation was assessed by a single-base elongation and ligation-based qPCR amplification method (SELECT) assay. Functions of MYH1G-AS were investigated through a primary myoblast and lentivirus/cholesterol-modified antisense oligonucleotide (ASO)-mediated animal model. To validate the interaction of MYH1G-AS with fibroblast growth factor 18 (FGF18) protein, RNA pull down and an RNA immunoprecipitation (RIP) assay were performed. Specifically, the interaction between FGF18 and SWI/SNF-related matrix-associated actin-dependent regulator of chromatin subfamily A member 5 (SMARCA5) protein was analyzed by coimmunoprecipitation (Co-IP) and a yeast two-hybrid assay.

**Results:**

A total of 45 differentially expressed (DE) lncRNAs, with DE ATAC-seq peaks in their promoter region, were classified as open chromatin-associated lncRNAs. A skeletal muscle-specific lncRNA (MSTRG.15576.9; MYH1G-AS), which is one of the open chromatin-associated lncRNA, was identified. MYH1G-AS transcription is coordinately regulated by transcription factors (TF) SMAD3 and SP2. Moreover, *SP2* represses *ALKBH5* transcription to weaken *ALKBH5*-mediated m^6^A demethylation of MYH1G-AS, thus destroying MYH1G-AS RNA stability. MYH1G-AS accelerates myoblast proliferation but restrains myoblast differentiation. Moreover, MYH1G-AS drives a switch from slow-twitch to fast-twitch fibers and causes muscle atrophy. Mechanistically, MYH1G-AS inhibits FGF18 protein stabilization to reduce the interaction of FGF18 to SMARCA5, thus repressing chromatin accessibility of the *SMAD4* promoter to activate the *SMAD4*-dependent pathway.

**Conclusions:**

Our results reveal a new pattern of the regulation of lncRNA expression at diverse levels and help expound the regulation of m^6^A methylation on chromatin status.

**Graphical Abstract:**

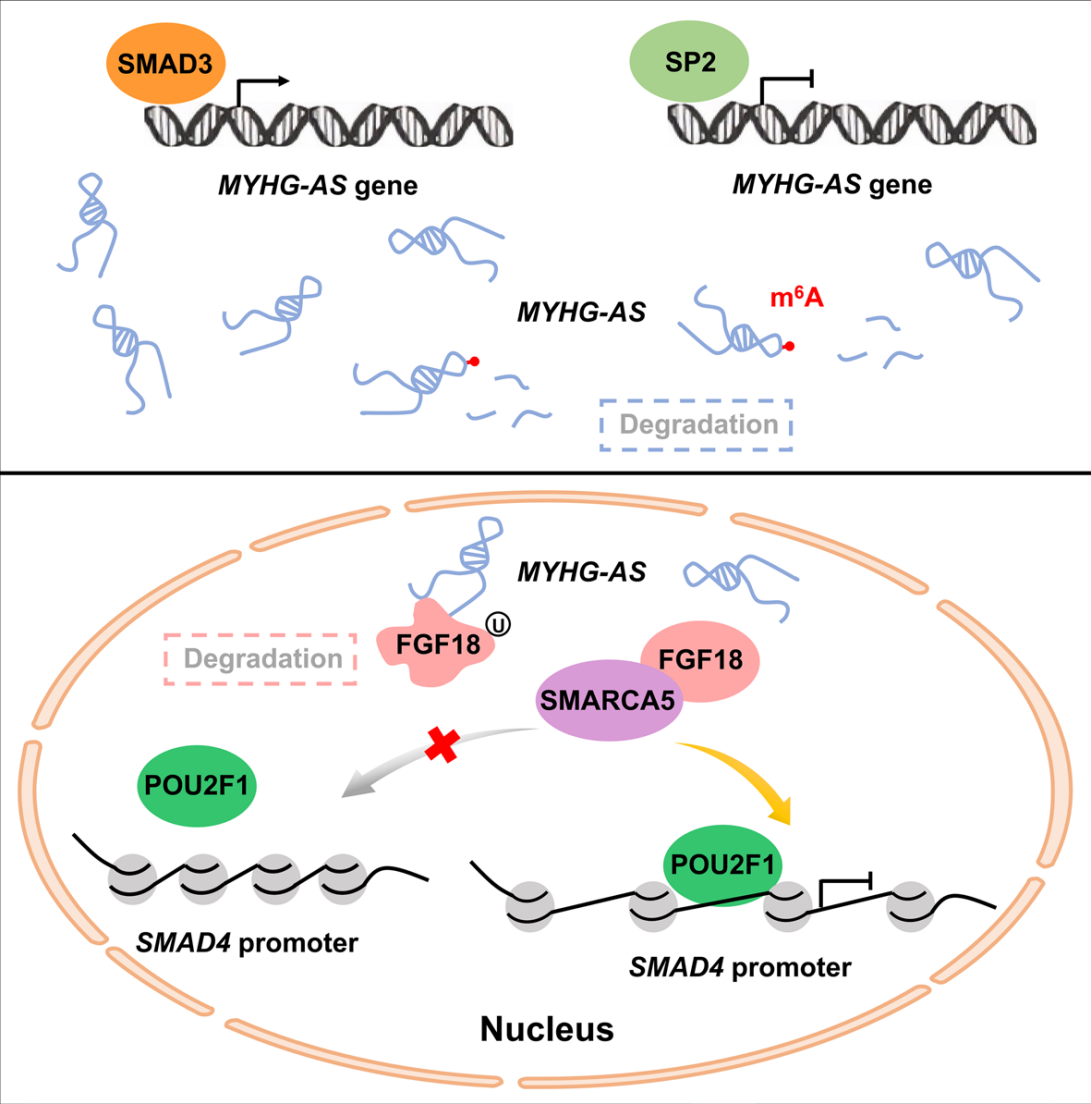

**Supplementary Information:**

The online version contains supplementary material available at 10.1186/s11658-023-00525-x.

## Background

Skeletal muscle consists of heterogeneous myofibers that are indispensable for locomotion, energy regulation, and posture maintenance [1]. After birth, skeletal muscle development is mainly regulated by changes in the type and size of myofibers. Notably, skeletal muscle development has been found to be essential for animal growth and health.

DNA transcription is dependent on chromatin opening. Dynamic changes in chromatin accessibility, which affect the binding of DNA-binding proteins such as TFs and RNA polymerases, regulated gene expression [2]. Interestingly, changes in chromatin accessibility are found to be closely relative to skeletal muscle development [3, 4].

As the most numerous transcripts in the genome, noncoding RNAs (ncRNAs) play important roles in epigenetic regulation [5, 6]. Recently, various ncRNAs have been found to be physically associated with chromatin, which are defined as chromatin-associated ncRNAs. As one of the ncRNAs, lncRNAs, are widely involved in epigenetic regulation of skeletal muscle development [4, 7–9]. Currently, only a fraction of lncRNAs have been well authenticated; however, they appear to interact with chromatin through diverse roles [10].

Here, to authenticate lncRNAs relative to skeletal muscle development and explore their transcriptional regulation, RNA-seq and ATAC-seq were performed. Based on these results, lncRNA MSTRG.15576.9 [named myosin, heavy chain 1G (*MYH1G*)-antisense transcript (MYH1G-AS)], was found to act as an open chromatin-associated lncRNA coordinately regulated by TF SMAD3 and SP2. Meanwhile, MYH1G-AS RNA stability was enhanced by *ALKBH5*-mediated m^6^A demethylation. Functional analysis revealed that MYH1G-AS regulates myogenesis, drives a switch from slow-twitch to fast-twitch fibers, and causes muscle atrophy. Mechanistically, MYH1G-AS inhibits FGF18 protein stabilization to reduce the interaction of FGF18 to SMARCA5, thus repressing chromatin accessibility of the *SMAD4* promoter to activate the *SMAD4*-dependent pathway. In general, our research discovers a chromatin-associated lncRNA that adjusts chromatin accessibility to regulate skeletal muscle development.

## Methods

### Ethics statement

All animal experimental protocols were conformed to “The Instructive Notions with Respect to Caring for Laboratory Animals”, issued by the Ministry of Science and Technology of the People’s Republic of China and approved by the Institutional Animal Care and Use Committee at the South China Agricultural University (approval ID: 2021c007).

### Animals and cells

Seven-week-old Xinghua female chickens, which were used for tissue separation, and 1-day-old chicks, which were used for animal experiment, were obtained from the Avian Farm of South China Agricultural University (Guangzhou, Guangdong, China).

Chicken primary myoblasts (CPMs) were isolated from the leg muscles of 11-embryonic-day-old chicken and cultured as previously described [11].

### RNA-seq

RNA-seq was performed using the strand-specific library construction method as previously described [12]. The purified library products were sequenced using an Illumina HiSeq™ 4000 and data were analyzed by Gene Denovo Biotechnology Co. (Guangzhou, China). For the reference genome mapping, the chicken genome GRCg6a was used. Raw data of RNA-seq were deposited in the Sequence Read Archive and Genome Sequence Archive (GSA) database under accession nos. PRJNA751251 and CRA008840.

### ATAC-seq

ATAC-seq was performed as previously described [13]. The Illumina HiSeq™ 4000 was used to sequence purified products. Bioinformatics analyses were conducted by Gene Denovo Biotechnology Co. (Guangzhou, China). The raw data of ATAC-seq were deposited in the GSA database under accession no. CRA008861.

### RNA extraction, cDNA synthesis, and real-time quantitative polymerase chain reaction (RT-qPCR)

RNA extraction, cDNA synthesis, and RT-qPCR was performed as previously described [14]. The primer pairs used for RT-qPCR and RT-PCR are listed in the Additional file 1: Table S1.

### Rapid amplification of complementary DNA ends (RACE)

RACE was performed by using a SMARTer RACE cDNA Amplification Kit (Clontech, Osaka, Japan). The primers used for RACE are listed in the Additional file 1: Table S1.

### RNA fluorescence in situ hybridization (FISH)

RNA FISH experiments were performed as previously described [11]. MYH1G-AS-specific FISH probes were modified by the cyanine dye Cy3.

### Vector construction and RNA oligonucleotides

Six potential Open Reading Frames (ORFs) of MYH1G-AS were cloned into the pcDNA3.1-3xFlag-C vector to construct Flag fusion protein expression vectors. Sequences of potential ORFs of MYH1G-AS are listed in the Additional file 1: Table S2.

For pGL3 luciferase reporter vector construction, wild type or mutated MYH1G-AS, *ALKBH5*, and *SMAD3* promoter fragments were cloned into the pGL3-basic vector (Promega, WI).

The MYH1G-AS full-length sequence, *SMAD3* coding sequence (NM_204475.1), *SP2* coding sequence (XM_025143997.2), *FGF18* coding sequence (NM_204714.1), *SMARCA5* coding sequence (XM_015276722.3), and *POU2F1* coding sequence (NM_205472.1) were cloned into the pcDNA-3.1 vector (Promega, Madison, WI) to generate overexpression vectors.

The MYH1G-AS full-length sequence was cloned into the overexpression lentiviral vector (pLVX-mCMV-ZsGreen-IRES-Puro; Addgene, Cambridge, MA). A short hairpin RNA against MYH1G-AS was designed and cloned into the knockdown lentiviral vector (pLVX-shRNA2-Puro; Addgene, Cambridge, MA).

The antisense oligonucleotide (ASO) and cholesterol-modified ASO against MYH1G-AS were designed and synthesized by Guangzhou RiboBio (Guangzhou, Guangdong, China). The small interfering RNAs (siRNA) against *SMAD3*, *SP2*, *FGF18*, *SMARCA5*, *SMAD4*, and *POU2F1* were also designed and synthesized.

The primer pairs for vector construction and the sequence of oligonucleotides are presented in the Additional file 1: Tables S1 and S3.

For transient cell transfection, a Lipofectamine 3000 reagent (Invitrogen, Carlsbad, CA) was used.

### Dual-luciferase reporter assay

Dual-luciferase reporter assays were performed as previously described [11, 15].

### ChIP assay

A chromatin immunoprecipitation (ChIP) assay was performed as previously described [15]. The primer pairs used for ChIP-qPCR are presented in the Additional file 1: Table S1.

### RNA dot blot, m^6^A RNA methylation quantification, and SELECT assay

Total RNA was denatured at 95 °C for 3 min and then dropped on a positively charged nylon membrane. Subsequently, crosslinking (2000 joules for 1 min) was performed by using a ultraviolet (UV) cross-linker. A specific m^6^A antibody (no. 56593S, 1:1000, Cell Signaling Technology, Boston) was used to detect the m^6^A methylation level

The EpiQuik™ m^6^A RNA Methylation Quantification kit (Colorimetric; P-9005, Epigentek, NY) was used for a total RNA m^6^A RNA methylation quantification assay.

SELECT assays were performed by using a Epi-SELECT™ m^6^A site-identification kit (R202106M-01, Epibiotek, Guangzhou, China). Relative SELECT products were calculated using the 2^−ΔΔCt^ method. The primers used for the SELECT assays are provided in the Additional file 1: Table S1.

### 5-ethynyl-2′-deoxyuridine (EdU), flow cytometry, and cell counting kit-8 (CCK-8) assay

EdU, flow cytometry, and CCK-8 assays were performed as previously described [14].

### Immunofluorescence (IF) and immunoblotting

IF was performed by using anti-MyHC (B103, 2.5 µg/mL, DSHB, IA), anti-FGF18 (bs-9762R, 1:200, Bioss, Beijing, China), or anti-SMARCA5 (bs-12653R, 1:100, Bioss, Beijing, China) as previously described [16]. A tyramide signal amplification kit (G1226, Servicebio, Wuhan, China) was used for fluorescent double-label staining.

Immunoblotting was performed as previously described [16]. The primary antibodies used in this study were anti-FLAG (14793, 1:1000, Cell Signaling Technology, MA), anti-MYF5 (bs-6936R, 1:500, Bioss, Beijing, China), anti-MYOD1 (M6190, 1:1000, Merck, NJ), anti-MYOG (orb6492, 1:500, Biorbyt, Cambridge, UK), anti-MYH1A (F59, 0.5 µg/mL, DSHB, IA), anti-MYH7B (S58, 0.5 µg/mL, DSHB, IA), anti-FBXO25 (LS-C31927, 1.25 µg/mL, LifeSpan Biosciences, WA), anti-FGF18 (bs-9762R, 1:500, Bioss, Beijing, China), anti-SMARCA5 (bs-12653R, 1:500, Bioss, Beijing, China), anti-Myc (AM926, 1:1000, Beyotime, Shanghai, China), anti-SMAD4 (bs-23966R, 1:500, Bioss, Beijing, China), anti-phosphorylated (p-) SMAD2 (bs-3419R, 1:500, Bioss, Beijing, China), anti-p-SMAD3 (bs-3425R, 1:500, Bioss, Beijing, China), and anti-β-Tubulin (A01030, 1:10,000, Abbkine, Wuhan, China). The goat anti-mouse IgG HRP (A21010, 1:10,000, Abbkine, Wuhan, China) and the goat anti-rabbit IgG HRP (A21020, 1:10,000, Abbkine, Wuhan, China) were used as a secondary antibody. Stability of the β-tubulin protein was judged by using other housekeeping genes (such as GAPDH and β-actin) (Additional file 1: Fig. S1). Raw images of western blot are shown in the Additional file 2.

### Lentivirus production and animal model construction

Lentivirus production and viral titer determination were performed as previously described [14].

Two groups [(a) Lv-MYH1G-AS and Lv-NC and (b) Chol-ASO-MYH1G-AS and Chol-ASO-NC] were randomly divided from 1-day-old chick. In brief, three intramuscular doses of gastrocnemius muscle (at days 1, 7, and 14) were performed with lentivirus (10^6^ titers) or cholesterol modified-ASO (40 nmol). The infected gastrocnemius muscle samples were collected 21 days after the first injection.

### Mitochondrial DNA (mtDNA) content, mitochondrial membrane potential, and reactive oxygen species (ROS) concentration assay

mtDNA content, mitochondrial membrane potential, and ROS concentration were measured as previously described [8]. The primers used to quantify the amount of mtDNA are list in the Additional file 1: Table S1.

### Central carbon metabolic profiling

A central carbon metabolic profiling assay was performed by using MYH1G-AS knockdown gastrocnemius samples (*n* = 7) as previously described [8].

The Cluster3.0 software was used for metabolic hierarchical clustering analysis (HCA).

### Metabolite and enzyme activities assays

The content of glycogen and enzyme activity of lactic dehydrogenase (LDH) and succinate dehydrogenase (SDH) were detected as previously described [8].

### ATPase staining, immunohistochemistry (IHC), and hematoxylin and eosin (HE) staining

ATPase staining, IHC, and HE staining were performed as previously described [14]. For IHC, anti-MYH1A (F59, 1:100, DHSB, IA) and anti-MYH7B (S58, 1:300, DHSB, IA) were used to label the signal.

For the measurement of cross-sectional area (CSA) of the myofiber, at least 60 myofibers were randomly selected in each replicate. More than 360 myofibers in total were subjected to statistical analysis of the frequency distribution of fiber CSA.

### RNA pull-down and RIP assays

RNA pull-down and RIP assays were performed as previously described [8]. For the RIP assay, the antibody anti-FLAG (14793, 1:50, Cell Signaling Technology, MA) was used.

### Co-IP assay

Co-IP assays were performed by using the Immunoprecipitation Kit with Protein A + G Agarose Gel (P2197S, Beyotime, Shanghai, China). For Co-IP assays, anti-FLAG (14793, 1:50, Cell Signaling Technology, MA) and anti-Myc (AM926, 1:100, Beyotime, Shanghai, China) were used.

### Yeast two-hybrid assay

A yeast two-hybrid assay was performed as described in the Yeast Protocols Handbook (Clontech, Japan). The *FGF18* coding sequence, except signal peptide, was cloned into the prey vector (pGBKT7, Clontech, Japan). For the prey vector construction, the *SMARCA5* coding sequence was cloned into the pGADT7 vector (Clontech, Japan).

###  Statistical analysis

In this study, each experiment was conducted at least three times, and the results appeared as mean ± standard error of the mean (SEM). An independent sample *t*-test or analysis of variance (ANOVA), followed by Dunnett’s test, were used to test the statistical significance of the data. The test type and *P*-values, when applicable, are presented in the figure legends.

## Results

### Dynamic transcriptional and chromatin accessibility landscapes of different myofibers in chicken

**Fig. 1:**
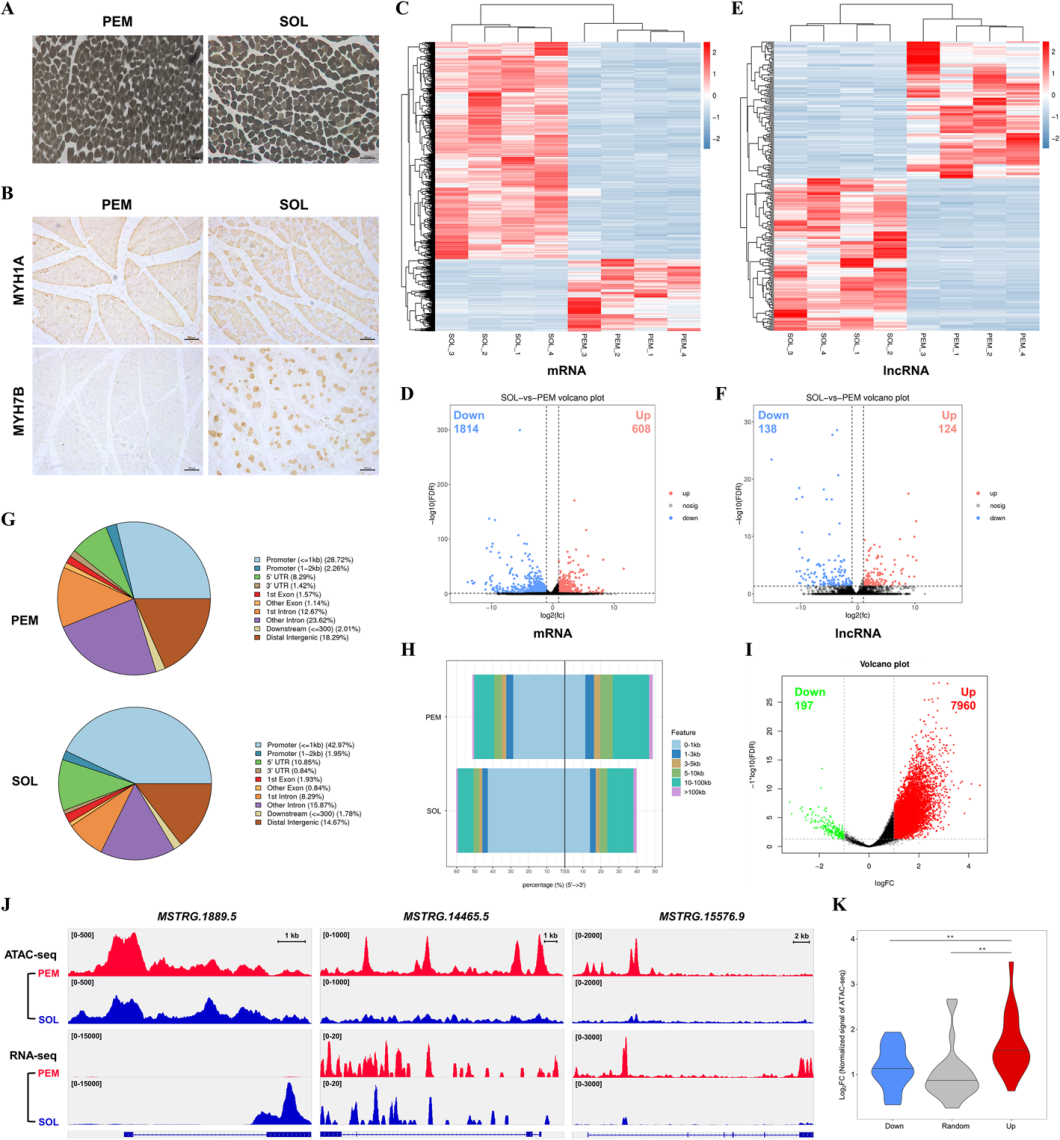
Overview of transcriptome and chromatin accessibility profile in different myofibers. **A**, **B** ATPase staining (**A**) and IHC analysis (**B**) of PEM and SOL in 7-week-old Xinghua chicken. **C**–**F** Heatmaps (**C** and **E**) and volcano plots (**D** and **F**) of differentially expressed mRNAs (**C**, **D**) and lncRNAs (**E**, **F**). **G** Pie charts showing the distribution of ATAC-seq peaks across the genome. The different colors represent different genomic regions. **H** Accumulated barplot showing the feature distribution of ATAC-seq peaks around the TSS. **I** Volcano plots of differentially expressed ATAC-seq peaks. **J** Gene view of coverage of lncRNA and chromatin-accessibility footprint reads on selected genes. Gene structure is diagrammed at the bottom. **K** Violin plot showing the changes in ATAC-seq intensity around the TSS of open chromatin-associated lncRNAs and randomly selected nondifferentially expressed lncRNAs. Results are shown as mean ± SEM. In panel **K**, the statistical significance of differences between means was assessed using an independent sample *t*-test. (**P < 0.01)

Heterogeneous myofibers are the primary components of skeletal muscle, with different metabolic and physiological characteristics [17]. Using ATPase staining, we found that the pectoralis major (PEM) entirely consists of fast-twitch fibers, while the soleus (SOL) has a higher proportion of slow-twitch myofibers (Fig. 1A). Analogously, immunohistochemical results showed that MYH1A (fast-twitch marker protein) was expressed in all myofibers of PEM, whereas MYH1A and MYH7B (slow-twitch marker protein) proteins were expressed in SOL (Fig. 1B), suggesting that there was a difference in myofiber composition between PEM and SOL.

To authenticate the lncRNAs relative to skeletal muscle development, RNA-seq was performed. In total, 2422 DE genes and 262 DE lncRNAs were identified between PEM and SOL (Fig. 1C–F; Additional file 3: Table S4, and Additional file 4: Table S5). These DE genes were enriched in biological processes including cellular process, metabolic process, and single-organism process and involved in pyruvate metabolism, regulation of actin cytoskeleton, and the cGMP-PKG signaling pathway, which are related to skeletal muscle development (Additional file 1: Fig. S2A, B). ATAC-seq was further performed to reveal the dynamic landscape of chromatin accessibility in different myofibers. Compared with SOL, higher mapping rates of peak to genome (0.77% in PEM and 0.33% in SOL) and more ATAC signals at the transcriptional start site (TSS) were found in PEM (Additional file 1: Fig. S3A, B), declaring that PEM has a greater chromatin accessibility. The genomic feature distribution showed that more than 67% of ATAC-seq peaks in PEM are in introns (36.29%) and promoters (30.98%), and the distance distribution of those peaks to TSS is relatively uniform (Fig. 1G, H). In SOL, nearly half of (44.89%) ATAC-seq peaks are enriched at promoters and concentrated in the 5′ upstream of TSS (Fig. 1G-H). Furthermore, 7960 differential ATAC-seq peaks were discovered (Fig. 1I; Additional file 5: Table S6). Gene ontology (GO) enrichment analysis found that genes associated with differential peaks were mainly enriched in cellular process, single-organism process, and metabolic process (Additional file 1: Fig. S2C). Moreover, these genes participated in the insulin signaling pathway, autophagy, and FoxO signaling pathway (Additional file 1: Fig. S2D).

A total of 45 DE lncRNAs, with DE ATAC-seq peaks in their promoter region (within 2 kb upstream of TSS), were classified as open chromatin-associated lncRNAs (Fig. 1J; Additional file 6: Table S7). Importantly, open chromatin-associated upregulated lncRNAs have a higher ATAC-seq intensity around the TSS (Fig. 1K), indicating that the change in chromatin accessibility leads to a change in lncRNA expression.

### MYH1G-AS is a skeletal muscle-specific lncRNA that is coordinately modulated by SMAD3 and SP2

**Fig. 2:**
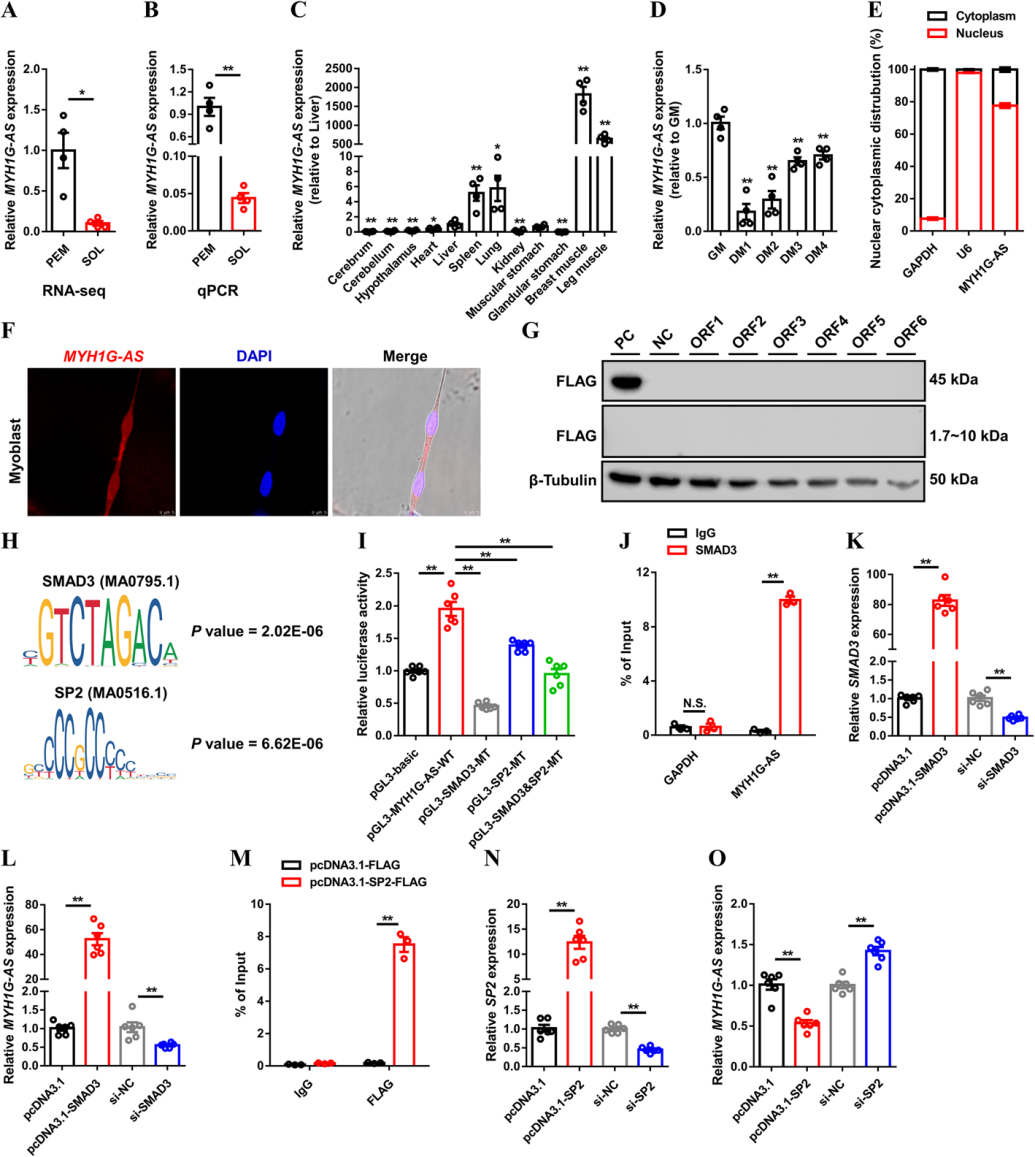
MYH1G-AS is a skeletal muscle-specific lncRNA that is coordinately regulated by SMAD3 and SP2. **A**, **B** Relative MYH1G-AS expression in PEM and SOL of 7-week-old Xinghua chicken detected by RNA-seq (**A**) and qPCR (**B**). **C** Tissue expression profiles of MYH1G-AS. **D** Relative MYH1G-AS expression during CPM proliferation and differentiation. **E** The distribution of MYH1G-AS in the cytoplasm and nucleus of CPMs determined by qPCR. Glyceraldehyde-3-phosphate dehydrogenase (*GAPDH*) and *U6* serve as cytoplasmic and nuclear localization controls, respectively. **F** RNA in situ hybridization of MYH1G-AS in CPM. Special FISH probes against MYH1G-AS were modified by Cy3 (red). The nucleus was stained by DAPI (blue). **G** Western blot analysis of the coding ability of MYH1G-AS. CPMs transfected with β-actin were used as a positive control (PC) and untransfected CPMs were used as a negative control (NC). **H** Significantly enriched TFs in MYH1G-AS upstream the ATAC-seq peak was predicted by MEME suite. **I** Transcriptional activity of MYH1G-AS upstream ATAC-seq peak. **J** ChIP analysis of the binding capacity of SMAD3 to the MYH1G-AS promoter. **K**, **L** Relative *SMAD3* (**K**) and MYH1G-AS (**L**) expression after *SMAD3* overexpression or interference. **M** ChIP analysis of the binding capacity of SP2 to MYH1G-AS promoter. **N**, **O** Relative *SP2* (**N**) and MYH1G-AS (**O**) expression with *SP2* overexpression or inhibition. Results are presented as mean ± SEM. In panels **A**–**D** and **I**–**O**, statistical significance of differences between means was assessed using an independent sample *t*-test. (*P < 0.05; **P < 0.01)

MYH1G-AS, which is one of the open chromatin-associated lncRNAs highly expressed in PEM (Fig. 1J, Fig. 2A, B; Additional file 6: Table S7), was selected as a candidate. First, RACE assay was performed to acquire a MYH1G-AS full-length sequence (Additional file 1: Fig. S4A). As an antisense transcript of *MYH1G*, MYH1G-AS was 1221 nt long and mainly conserved in Aves (Additional file 1: Fig. S4B, C). We further investigated the expression pattern of MYH1G-AS and found that MYH1G-AS was specifically highly expressed in skeletal muscle and downregulated during myogenic differentiation (Fig. 2C, D). A cell-fractionation assay and in situ RNA hybridization demonstrated that MYH1G-AS is mainly present in the nucleus (Fig. 2E, F). Moreover, the coding potential of MYH1G-AS was verified, and the result of western blotting suggested that MYH1G-AS was without protein-encoding potential (Fig. 2G).

The MEME suite was used to analyze a significant motif and predict its significantly enriched TFs in the MYH1G-AS upstream DE ATAC-seq peak. SMAD3 and SP2, which could potentially bind to the promoter of MYH1G-AS, were found (Fig. 2H). The dual-luciferase reporter assays showed that mutation of the SMAD3 potential binding site decreased the transcription activity of the MYH1G-AS promoter, while the transcription activity of the MYH1G-AS promoter was increased with mutation in the SP2 potential binding site (Fig. 2I). The binding of SMAD3 and SP2 to the MYH1G-AS promoter was also verified by ChIP assays (Fig. 2J and M). Furthermore, *SMAD3* overexpression strongly upregulated MYH1G-AS expression, while MYH1G-AS expression was reduced with *SP2* overexpression (Fig. 2K, L and N, O). On the contrary, *SMAD3* interference suppressed MYH1G-AS expression, while MYH1G-AS expression was promoted after *SP2* inhibition (Fig. 2K, L and N, O). Collectively, these data revealed that SMAD3 and SP2 coordinately regulate the transcription activity of MYH1G-AS.

### ALKBH5-mediated m^6^A demethylation enhances RNA stability of MYH1G-AS

**Fig. 3:**
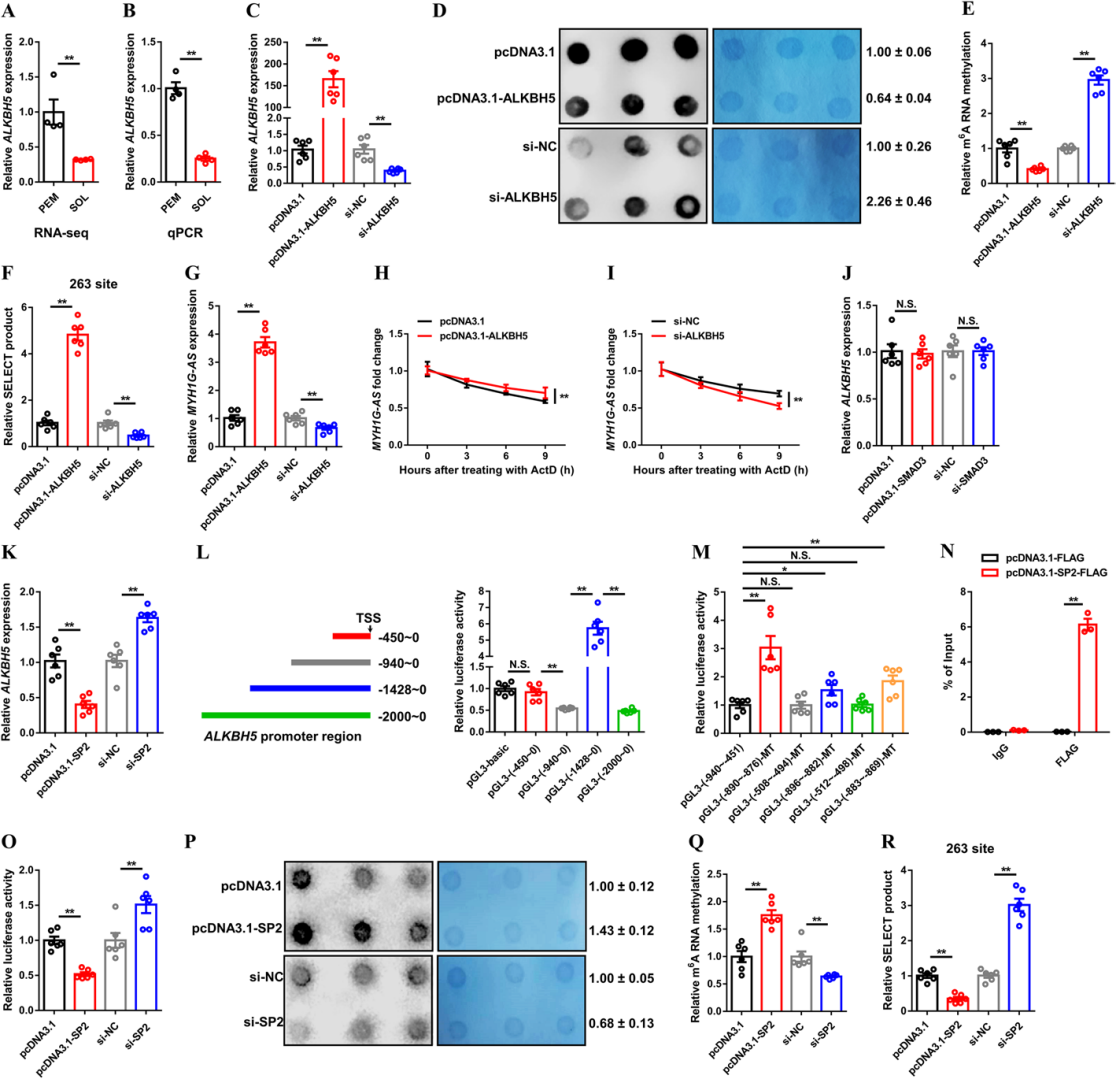
ALKBH5-mediated m^6^A demethylation maintains RNA stability of MYH1G-AS. **A**, **B** Relative *ALKBH5* expression in PEM and SOL of 7-week-old Xinghua chicken detect by RNA-seq (**A**) and qPCR (**B**). **C**–**G** Relative *ALKBH5* expression (**C**), RNA dot blot assay (**D**), relative m^6^A methylation level (**E**), relative SELECT product at the 263 site of MYH1G-AS (**F**), and relative MYH1G-AS expression (**G**) after *ALKBH5* overexpression or interference. **H**, **I** MYH1G-AS RNA stability assay after *ALKBH5* overexpression (**H**) and inhibition (**I**). **J** Relative *ALKBH5* expression after *SMAD3* (**J**) and *SP2* (**K**) overexpression or interference. **L** Left: Schematic of four truncated *ALKBH5*-promoter constructs used for luciferase assays. Right: Dual-luciferase reporter assays of four reporter constructs. **M** The transcriptional activity of the *ALKBH5* core promoter region. **N** ChIP analysis of the binding capacity of SP2 to *ALKBH5* promoter. **O** The transcriptional activity of *ALKBH5* core promoter region after *SP2* overexpression or knockdown. **P**–**R** RNA dot blot assay (**P**), relative m^6^A methylation level (**Q**), and relative SELECT product at the 263 site of MYH1G-AS (**R**) with *SP2* overexpression or interference. Results are showed as mean ± SEM. In panels **A**–**C**, **E**–**O**, and **Q**, **R** statistical significance of differences between means was assessed using an independent sample *t*-test. (*P < 0.05; **P < 0.01; NS, no significant difference)

As the richest class of methylation modifications, m^6^A is well known to participate in multiple regulatory processes of RNA metabolism, widely regulating skeletal muscle development [18, 19]. Given that ALKBH5, which is a m^6^A demethylase, was highly expressed in PEM (Fig. 3A, B), we analyzed whether *ALKBH5* participates in the regulation of MYH1G-AS expression by mediating its m^6^A demethylation. As expected, *ALKBH5* overexpression induced m^6^A demethylation, whereas the m^6^A methylation level was upregulated with the *ALKBH5* interference (Fig. 3C–E). The potential m^6^A modification sites on MYH1G-AS were further predicted by using the SRAMP (http://www.cuilab.cn/sramp) software and verified by SELECT assays. A total of seven potential m^6^A modification sites were found, and the SELECT product at the 263 site was increased with *ALKBH5* overexpression (Fig. 3F and Additional file 1: Fig. S5A–G). Conversely, *ALKBH5* interference promoted the m^6^A methylation at the 263 site of MYH1G-AS (Fig. 3F). Moreover, *ALKBH5* overexpression facilitated the expression and RNA stability of MYH1G-AS, whereas MYH1G-AS expression was inhibited and MYH1G-AS RNA stability was destroyed after *ALKBH5* interference (Fig. 3G–I), suggesting that *ALKBH5* promotes MYH1G-AS expression by inducing m^6^A demethylation at the 263 site of MYH1G-AS.

TFs are well known to recognize specific motifs in promoters, thereby widely regulating the transcription and expression of its target genes. *ALKBH5* expressions were detected after *SMAD3* or *SP2* overexpression and interference. Overexpression or interference of *SMAD3* did not change the *ALKBH5* expression (Fig. 3J). Interestingly, *SP2* overexpression repressed *ALKBH5* expression, whereas *ALKBH5* expression was upregulated with *SP2* inhibition (Fig. 3K). To explore the potential binding site of SP2 on the promoter of *ALKBH5*, promoter truncation experiments were performed. Luciferase activities in the − 940 ~ 0 region and − 2000 ~ 0 regions were significantly reduced (Fig. 3L), indicating that there are binding sites for silent elements between − 940 to − 450 and − 2000 to − 1428 region. The potential binding sites for SP2 in these regions were further predicted by JASPAR (https://jaspar.genereg.net/) software and verified by several dual-luciferase reporter assays (Fig. 3M). The transcription activity of *ALKBH5* was improved with the mutation of the − 896 region to the − 869 (− 890 to − 876, − 896 to − 882 and − 883 to − 869) region (Fig. 3M), which is potential bound to SP2. The binding of SP2 was also confirmed by the ChIP assay (Fig. 3N). *SP2* overexpression suppressed *ALKBH5* transcription, whereas *ALKBH5* transcription was increased with *SP2* interference (Fig. 3O). Furthermore, *SP2* promoted m^6^A methylation and reduced SELECT product at the 263 site of MYH1G-AS (Fig. 3P–R and Additional file 1: Fig. S5H–M), which explains that *SP2* represses the transcription of *ALKBH5* to reinforce the inhibition of MYH1G-AS expression.

### MYH1G-AS regulates myogenesis, drives a switch from slow-twitch to fast-twitch fibers, and causes muscle atrophy

**Fig. 4:**
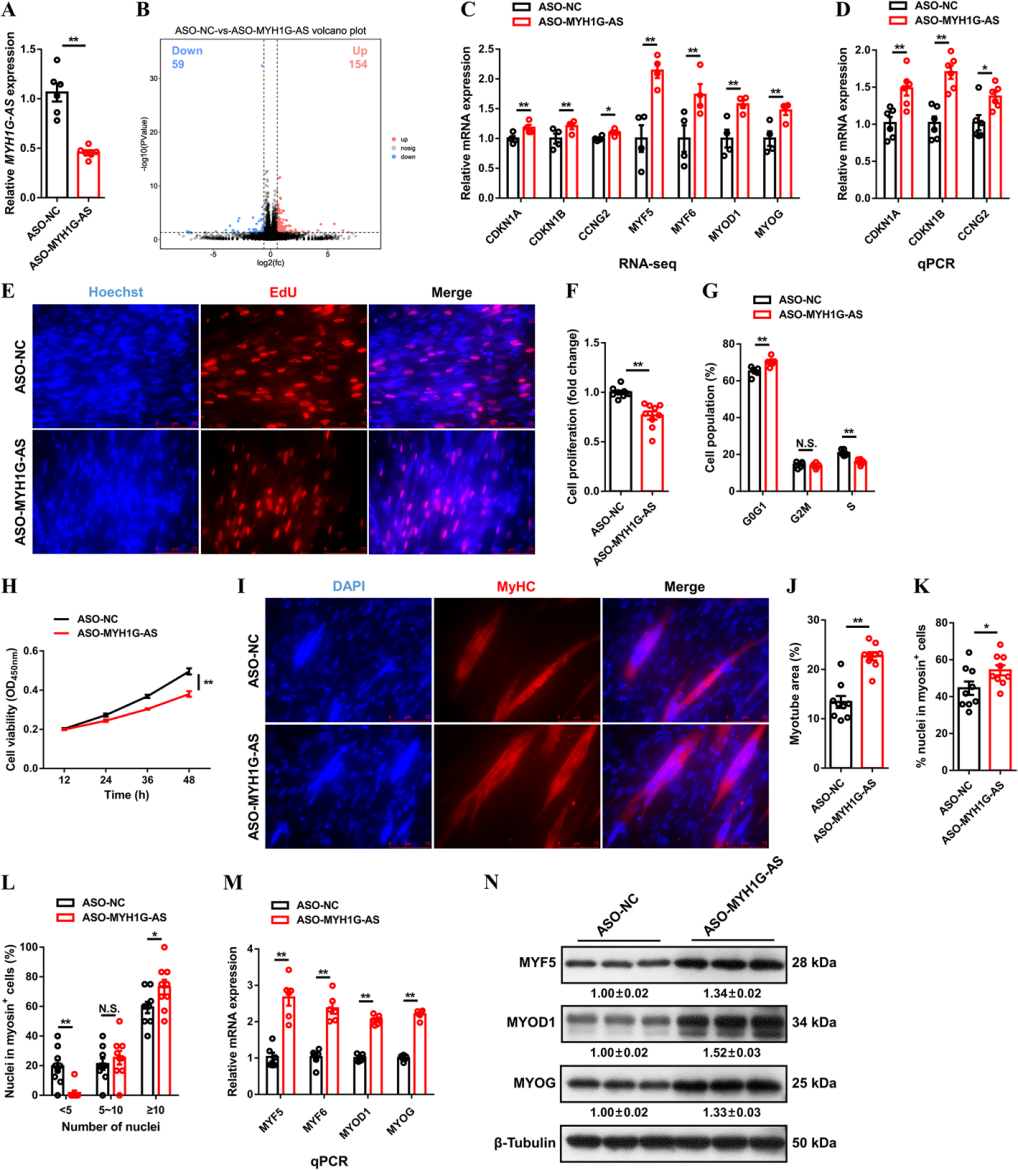
MYH1G-AS facilitates myoblast proliferation but inhibits myogenic differentiation. **A** Relative MYH1G-AS expression with MYH1G-AS interference in CPMs. **B** Volcano plots of differentially expressed genes between the control group and MYH1G-AS interference. **C** Relative mRNA expressions of several differentially expressed genes after MYH1G-AS interference detected by RNA-seq. **D**−**M** Relative mRNA expressions of several cell cycle-inhibiting genes (**D**), EdU proliferation assay (**E**), proliferation rate of myoblasts (**F**), cell cycle analysis (**G**), CCK-8 assay (**H**), MyHC immunostaining (**I**), myotube area (**J**), differentiation index (**K**), myoblast fusion index (**L**), and relative mRNA (**M**) and protein (**N**) expression levels of myoblast differentiation marker genes with MYH1G-AS inhibition in vitro. In panel **N**, the numbers shown below the bands were folds of band intensities relative to the control. Band intensities were quantified by ImageJ and normalized to β-tubulin. Results are presented as mean ± SEM. In panels **A**, **C**, **D**, **F**−**H**, and **J**−**M**, statistical significance of differences between means was assessed using an independent sample *t*-test. (*P < 0.05; **P < 0.01)

To screen target genes regulated by MYH1G-AS and explore its potential functions, RNA-seq was performed after MYH1G-AS interference (Fig. 4A). In total, 213 genes were identified as being DE between the control group and MYH1G-AS interference CPMs (*P* < 0.05; |FC| > 1.5) (Fig. 4B; Additional file 7: Table S8). According to GO enrichment analysis, DE genes were mainly related to cellular processes, single-organism processes, and biological regulation (Additional file 1: Fig. S6A). KEGG pathway analysis indicated that cytokine–cytokine receptor interactions, signaling pathways regulating pluripotency of stem cells, and the TGF-β signaling pathway are the top three enriched pathways of those DE genes (Additional file 1: Fig. S6B).

Several genes, including *CDKN1A*, *CDKN1B*, *CCNG2*, *MYF5*, *MYF6*, *MYOD1* and *MYOG*, which are involved in cell cycle and myogenic differentiation, were found to be DE after MYH1G-AS interference (Fig. 4C). In addition, considering that MYH1G-AS was downregulated during myoblast differentiation (Fig. 1D), we study the function of MYH1G-AS in myogenesis. Cell cycle-inhibiting genes, such as *CDKN1A*, *CDKN1B*, and *CCNG2*, were upregulated with MYH1G-AS interference (Fig. 4D). EdU staining showed that MYH1G-AS interference decreased EdU incorporation and impeded proliferation of myoblasts (Fig. 4E, F). Moreover, flow cytometric analysis and CCK-8 assay showed that MYH1G-AS interference led to fewer S phase cells and downregulated myoblast viability (Fig. 4G, H). IF staining was further performed, and the result showed that MYH1G-AS inhibition increased the total areas of myotubes, increased the differentiation index, and induced the formation of myotubes (Fig. 4I−L). Besides, myoblast differentiation-related genes, such as *MYF5*, *MYF6*, *MYOD1*, and *MYOG*, were upregulated after MYH1G-AS interference (Fig. 4M, N). Conversely, opposite results were observed by MYH1G-AS overexpression (Additional file 1: Fig. S7A-L), illustrating that MYH1G-AS facilitates myoblast proliferation but hinders myogenic differentiation.

**Fig. 5:**
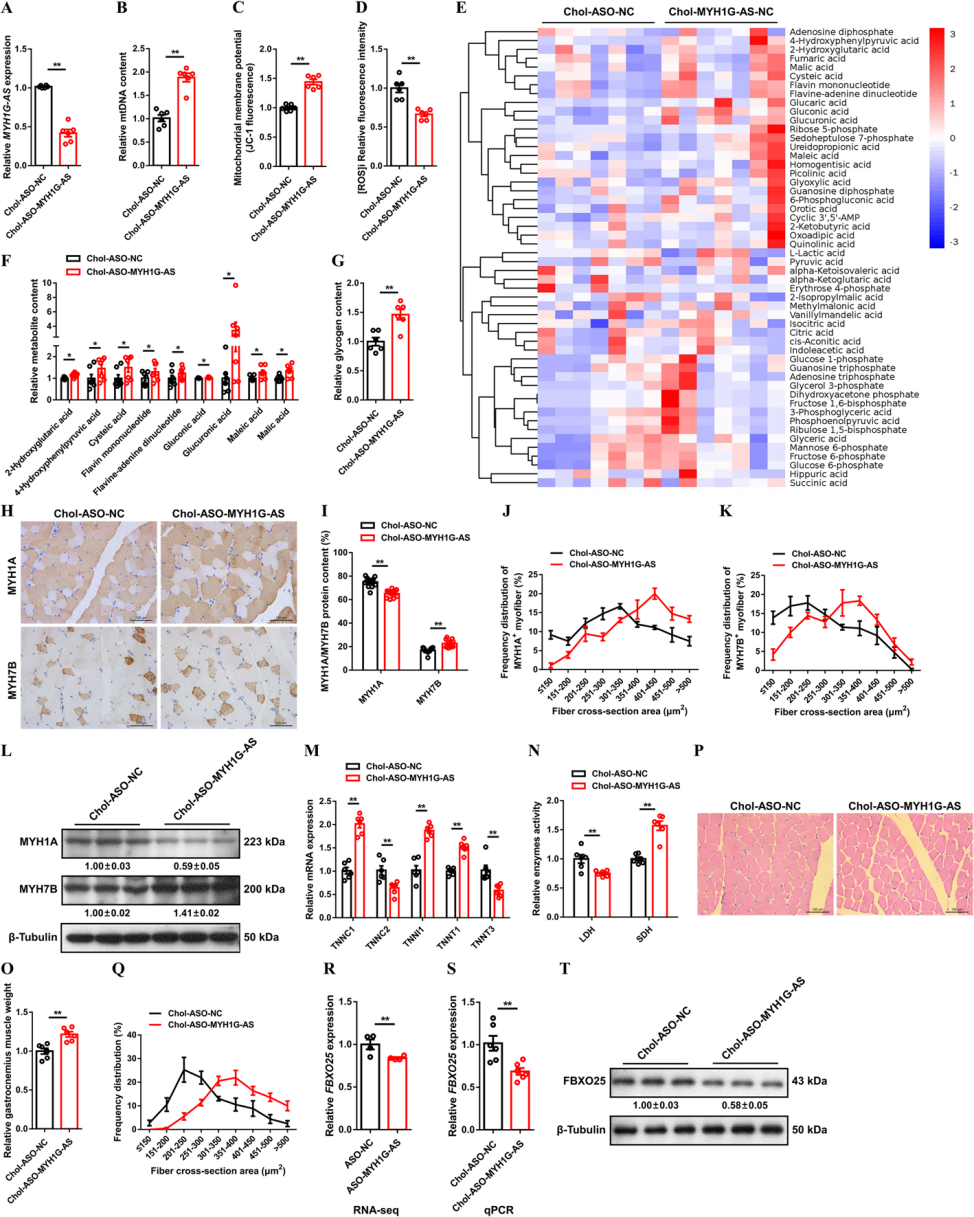
MYH1G-AS modulates skeletal muscle metabolism to activate fast-twitch muscle phenotype and induces muscle atrophy. **A**−**D** Relative MYH1G-AS expression (**A**), relative mtDNA content (**B**), mitochondrial membrane potential (**C**), and intracellular ROS ([ROS]i) (**D**) in gastrocnemius with MYH1G-AS knockdown. **E** HCA of metabolites in gastrocnemius after infected with Chol-ASO-MYH1G-AS or Chol-ASO-NC. The colors indicate the relative levels in control or MYH1G-AS knockdown group. **F** Relative metabolite content in MYH1G-AS knockdown gastrocnemius detected by central carbon metabolic profiling. **G**−**N** Relative glycogen content (**G**), IHC analysis (**H**), MYH1A/MYH7B protein content (**I**), frequency distribution of MYH1A^+^ (**J**) and MYH7B^+^ (**K**) myofiber CSA, relative protein expression of MYH1A and MYH7B (**L**), relative mRNA expression of several fast- and slow-twitch myofiber genes (**M**), relative enzymes activity of LDH and SDH (**N**), relative gastrocnemius muscle weight (**O**), H&E staining (**P**), and frequency distribution of fiber CSA (**Q**) in gastrocnemius with MYH1G-AS knockdown. **R** Relative mRNA expression of *FBXO25* after MYH1G-AS interference detected by RNA-seq. **S**, **T** Relative mRNA (**S**) and protein (**T**) expression of *FBXO25* after MYH1G-AS knockdown. In panel **L** and **T**, the numbers shown below the bands were folds of band intensities relative to control. Band intensities were quantified by ImageJ and normalized to β-tubulin. Results are shown as mean ± SEM. In panels **A**−**D**, **F**, **G**, **I**, **M**−**O**, and **R**, **S**), the statistical significance of the differences between means was assessed using paired *t*-tests. (*P < 0.05; **P < 0.01)

To verify the function of MYH1G-AS in vivo, cholesterol modified, ASO-mediated MYH1G-AS knockdown (Chol-ASO-MYH1G-AS) and lentivirus-mediated MYH1G-AS overexpression (Lv-MYH1G-AS) animal models were conducted (Fig. 5A and Additional file 1: Fig. S8A). MYH1G-AS knockdown increased mtDNA content and improved mitochondrial membrane potential (Fig. 5B, C), and ROS production was reduced with MYH1G-AS knockdown (Fig. 5D). Inversely, mtDNA content was decreased and mitochondrial function was impeded after MYH1G-AS overexpression (Additional file 1: Fig. S8B−D). Comparative metabolome analysis found that MYH1G-AS knockdown increased tricarboxylic acid cycle metabolite such as malic acid (Fig. 5E, F; Additional file 8: Table S9). The accumulation of glycogen was facilitated after MYH1G-AS knockdown (Fig. 5G). More importantly, results of immunohistochemistry and western blot showed that MYH1G-AS knockdown suppressed the MYH1A protein level but promoted the expression level of MYH7B protein, as well as aggrandized the CSA of MYH1A^+^ and MYH7B^+^ myofibers (Fig. 5G−L). In addition, MYH1G-AS knockdown promoted expressions of slow-twitch myofiber genes, such as *TNNC1*, *TNNI1*, and *TNNT1*, and repressed expressions of fast-twitch myofiber genes, such as *TNNC2* and *TNNT3* (Fig. 5M). The activity of LDH was repressed while SDH activity was facilitated after MYH1G-AS knockdown (Fig. 5N). Opposite results were observed with MYH1G-AS overexpression (Additional file 1: Fig. S8E−L), indicating that MYH1G-AS suppresses mitochondria biogenesis to modulate skeletal muscle metabolism, thus activating the fast-twitch muscle phenotype.

Recent studies have found that muscle remodeling can induce muscle hypertrophy or atrophy by modulating muscle metabolism [20]. Here, muscle mass was increased and the CSA of myofibers were enlarged after MYH1G-AS knockdown (Fig. 5O−Q). Conversely, MYH1G-AS overexpression reduced gastrocnemius mass and lessened the size of myofibers (Additional file 1: Fig. S8M−O). The ubiquitin–proteasome system (UPS) is well known to regulate skeletal muscle atrophy [21, 22]. *FBXO25*, which is an ubiquitin E3 ligase involved in UPS, was found downregulated after MYH1G-AS interference (Fig. 5R). In vivo, MYH1G-AS knockdown repressed *FBXO25* expression, whereas *FBXO25* expression was promoted after MYH1G-AS overexpression (Fig. 5S, T and Additional file 1: Fig. S8P, Q). Given that MYH1G-AS induced atrophy of both fast-twitch and slow-twitch myofibers, we conclude that MYH1G-AS-induced muscle atrophy should be attributed to its regulation of *FBXO25*, rather than induced by myofiber remodeling.

### MYH1G-AS binds with FGF18 to inhibit FGF18 protein stabilization

**Fig. 6:**
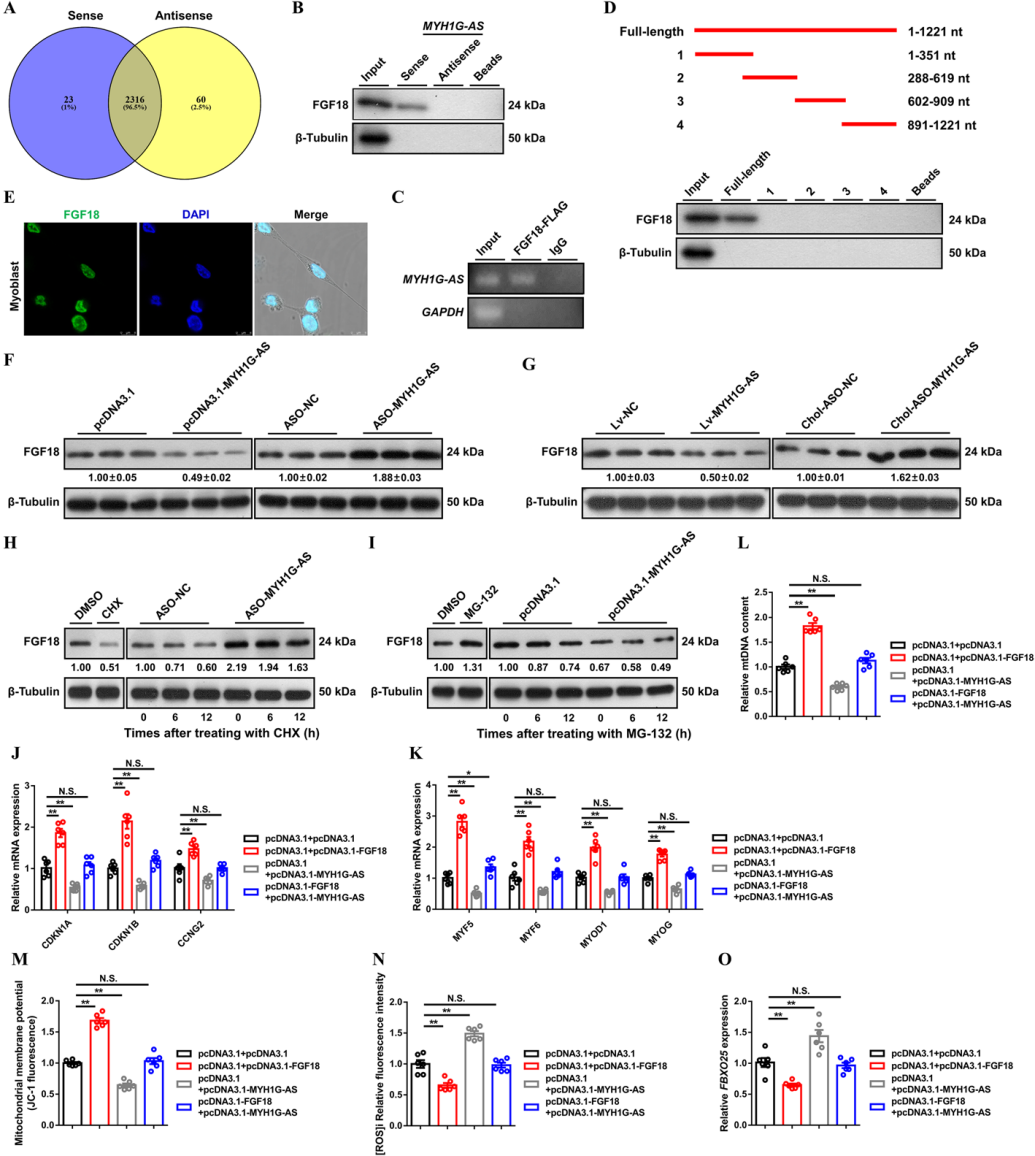
MYH1G-AS interacts with FGF18 to destroy FGF18 protein stabilization. **A** Venn diagram showing the specific binding proteins of the MYH1G-AS sense strand or antisense strand. **B**, **C** The interaction of MYH1G-AS with FGF18 protein was determined by biotin-labeled RNA pulldown (**B**) and RIP (**C**) assays. **D** The binding of full-length and truncated MYH1G-AS with FGF18 protein was determined by RNA pulldown assay. **E** IF staining of FGF18 in CPM. **F**, **G** The protein expression level of FGF18 after MYH1G-AS overexpression or knockdown in vitro (**F**) and in vivo (**G**). **H** Left: FGF18 protein expression in myoblasts after dimethyl sulfoxide (DMSO) or cycloheximide (CHX; 25 µg/mL) treatment for 12 h. Right: FGF18 protein expression in the MYH1G-AS-knockdown myoblast was analyzed after incubation with CHX. **I** Left: FGF18 protein expression in myoblasts after DMSO or MG-132 (5 µmol/L) treatment for 12 h. Right: FGF18 protein expression in MYH1G-AS overexpressed myoblast was analyzed after incubated with MG-132. **J**−**O** Relative mRNA expressions of several cell cycle-inhibiting genes (**J**), relative mRNA expressions of myoblast differentiation marker genes (**K**), relative mtDNA content (**L**), mitochondrial membrane potential (**M**), [ROS]i (**N**), and relative mRNA expressions of *FBXO25* (**O**) induced by the listed nucleic acids in CPMs. In panel **F**−**I**, the numbers shown below the bands were folds of band intensities relative to the control. Band intensities were quantified by ImageJ and normalized to β-tubulin. Results are presented as mean ± SEM. In panels **J**–**O**, the statistical significance of differences between means was assessed using an independent sample *t*-test. (*P < 0.05; **P < 0.01)

To clarify the molecular mechanism of MYH1G-AS, *MYH1G* expression was first examined. *MYH1G* mRNA was not changed with MYH1G-AS overexpression or knockdown (Additional file 1: Fig. S9A, B). Next, an RNA pull-down assay was performed. A total of 23 proteins were identified by mass spectrometry, suggesting they specifically bind to MYH1G-AS sense transcript (Fig. 6A; Additional file 9: Table S10). FGF18, which is a member of the fibroblast growth factor family, was found to be a endogenous interacting protein (Fig. 6B). A RIP assay was performed, which confirmed this specially interaction (Fig. 6C). Only a full-length strand of MYH1G-AS could physically bind to FGF18 (Fig. 6D), hinting that the complete RNA structure is indispensable for their interaction. Similar to MYH1G-AS, cellular localization was confirmed by subcellular location annotation and IF staining (Fig. 6E and Additional file 1: Fig. S10A), explaining the interaction of MYH1G-AS and FGF18.

MYH1G-AS did not modulate *FGF18* mRNA expression (Additional file 1: Fig. S9C, D). Crucially, FGF18 protein expression was downregulated with MYH1G-AS overexpression, while FGF18 protein level was enhanced after MYH1G-AS knockdown (Fig. 6F, G). Furthermore, treatment with CHX (which is a protein biosynthesis inhibitor) decreased FGF18 protein expression (Fig. 6H). However, this degradation was relieved with MYH1G-AS knockdown (Fig. 6H). Proteasome inhibitor MG-132 was also used to elucidate the induction effect of MYH1G-AS on FGF18 protein degradation. As expected, the protein levels of FGF18 were upregulated, and the reduction of FGF18 protein levels with MYH1G-AS overexpression was rescued with MG-132 treatment (Fig. 6I). Given that MYH1G-AS induced *FBXO25* expression (Fig. 5R−T and Additional file 1: Fig. S8P, Q), we deduced that MYH1G-AS causes the ubiquitination of FGF18 to promote FGF18 degradation.

*FGF18* was highly expressed in skeletal muscle (Additional file 1: Fig. S10B), and the expression level of *FGF18* increased gradually with myogenic differentiation (Additional file 1: Fig. S10C). in vitro experiments were conducted to study the potential biological function of *FGF18* in myogenesis. An opposite result to MYH1G-AS was observed, suggesting that *FGF18* had an inverse biological function compared with MYH1G-AS (Additional file 1: Fig. S11A-R). Meanwhile, *FGF18* overexpression promoted expressions of cell cycle-inhibiting genes and myoblast differentiation-related genes, which neutralizes the effect of MYH1G-AS on myogenesis (Fig. 6J, K). The suppression of mitochondria biogenesis and promotion of *FBXO25* expression, which was induced by MYH1G-AS overexpression, were counteracted with *FGF18* overexpression (Fig. 6L−O), suggesting that *FGF18* mediates the function of MYH1G-AS.

### MYH1G-AS reduces the interaction of FGF18 to SMARCA5, thereby promoting *SMAD4* transcription and activating the *SMAD4*-dependent pathway

**Fig. 7:**
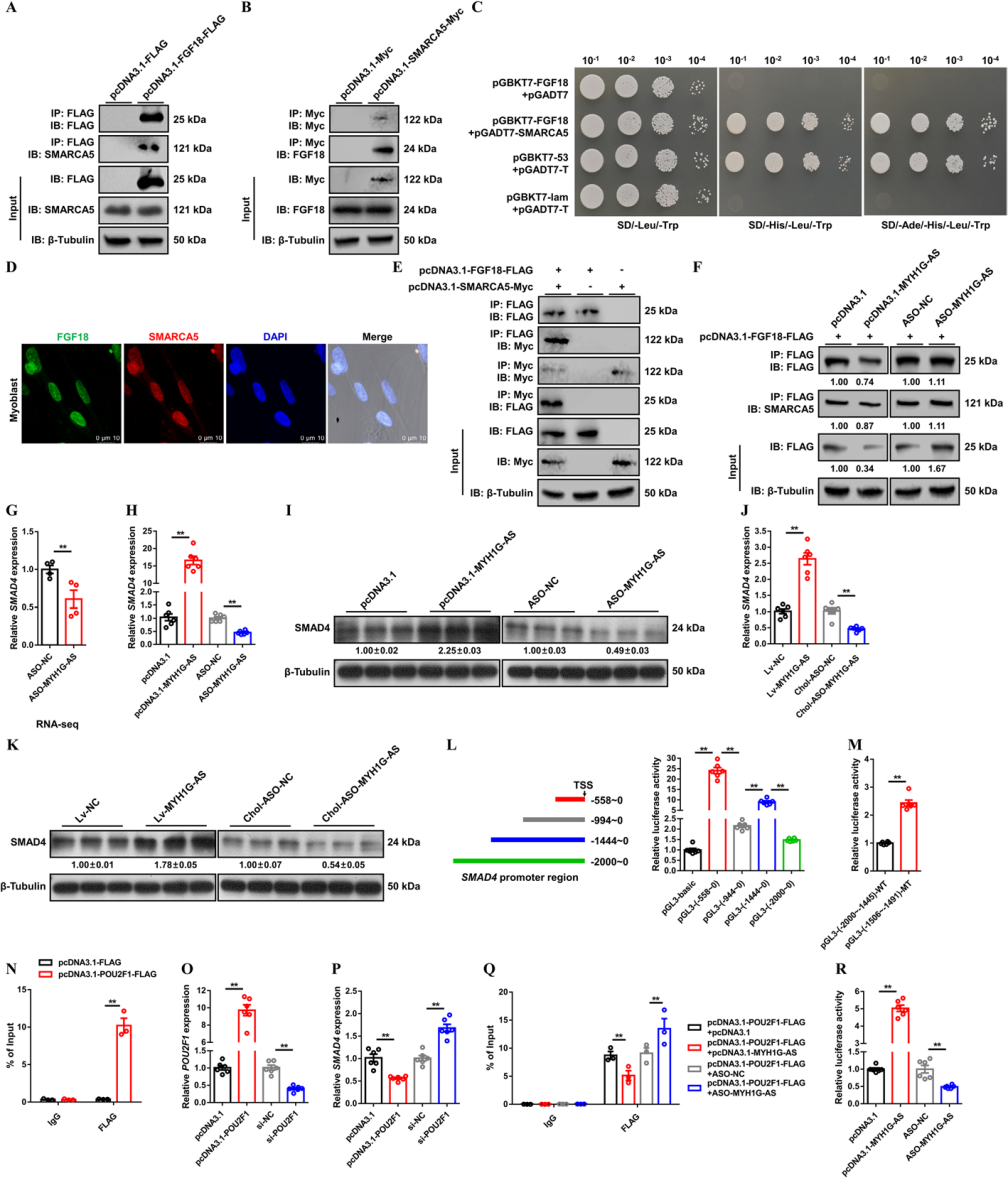
MYH1G-AS reduces interaction of FGF18 to SMARCA5, thereby promoting the transcription and expression of *SMAD4*. **A** The binding of FGF18 to the SMARCA5 protein was determined by Co-IP assay. **B** The binding of SMARCA5 to the FGF18 protein was determined by Co-IP assay. **C** The interaction between FGF18 and the SMARCA5 protein was determined by the yeast two-hybrid system. **D** IF staining of SMARCA5 and FGF18 in CPM. **E** The interaction between FGF18 and the SMARCA5 protein was determined by Co-IP assay. **F** Co-IP assay after cotransfection with pcDNA3.1-FGF18-FLAG and the listed nucleic acids. (**G**) Relative *SMAD4* expression after MYH1G-AS interference detected by RNA-seq. **H**−**K** Relative mRNA (**H** and **J**) and protein (**I** and **K**) expression levels of *SMAD4* with MYH1G-AS overexpression or knockdown in vitro (**H**, **I**) and in vivo (**J**−**K**). **L** Left: Schematic of four truncated *SMAD4* promoter constructs used for luciferase assays. Right: Dual-luciferase reporter assays of four reporter constructs. **M** The transcriptional activity of *SMAD4* core promoter region. **N** ChIP analysis of the binding capacity of POU2F1 to the *SMAD4* promoter. **O**, **P** Relative *POU2F1* (**O**) and *SMAD4* (**P**) expression after *POU2F1* overexpression or knockdown. **Q** The transcriptional activity of the *SMAD4* core promoter region after MYH1G-AS overexpression or knockdown. **R** ChIP analysis of the binding capacity of POU2F1 to the *SMAD4* promoter with MYH1G-AS overexpression or knockdown. In panels **F**, **I**, and **K**, the numbers shown below the bands were folds of band intensities relative to control. Band intensities were quantified by ImageJ and normalized to β-tubulin. Results are shown as mean ± SEM. In panels **G**, **H**, **J**, and **L**− **R**, the statistical significance of differences between means was assessed using an independent sample *t*-test. (**P < 0.01)

Considering that the molecular functional annotation of FGF18 is protein binding (Additional file 9: Table S10), Co-IP was performed to excavate its downstream interacting proteins. A total of 150 proteins were found to specifically interact with FGF18 by mass spectrometry (Additional file 10: Table S11). Among them, SWI/SNF-related matrix-associated actin-dependent regulator of chromatin subfamily A member 5 (SMARCA5), which belongs to the SWI/SNF family with remodeling activity [23, 24], was found. Co-IP and yeast two-hybrid assays confirmed the interaction between FGF18 and SMARCA5 (Fig. 7A−C). Nuclear localization of SMARCA5 was revealed by IF, which is similar to FGF18 (Fig. 7D). Moreover, Co-IP assays were further conducted after cotransfection with the FGF18-FLAG fusion expression vector and SMARCA5-MYC fusion expression vector, which also clearly stated that FGF18 specifically interacts with SMARCA5 (Fig. 7E). Neither MYH1G-AS nor *FGF18* could modulate the mRNA and protein expression of SMARCA5 (Additional file 1: Fig. S12A-F). Given that MYH1G-AS destroys the stabilization of FGF18 protein, we further explored whether MYH1G-AS hindered the interaction between FGF18 and the SMARCA5 protein. As expected, MYH1G-AS overexpression reduced the interaction between FGF18 and SMARCA5 by inhibiting FGF18 protein expression, whereas the interaction between FGF18 and SMARCA5 was heightened with MYH1G-AS interference (Fig. 7F).

SMAD family member 4 (SMAD4) is a pivotal signaling cascade of the TGF-β signaling pathway, which is widely involved in skeletal muscle development [25, 26]. Given that the TGF-β signaling pathway is one of the most enriched pathways in MYH1G-AS interference, myoblast and *SMAD4* was downregulated with MYH1G-AS inhibition (Fig. 7G and Additional file 1: Fig. S6B). We hypothesized that MYH1G-AS regulates *SMAD4* expression and modulates the *SMAD4*-dependent pathway. Both in vivo and in vitro, MYH1G-AS overexpression upregulated mRNA and protein expression of *SMAD4*, while *SMAD4* expression was suppressed with MYH1G-AS knockdown (Fig. 7H–K). Phosphorylated SMAD2 and SMAD3 have been reported to bind SMAD4 to participate in the TGF-β signaling pathway [27]. We also detected the phosphorylation levels of the SMAD2 and SMAD3 proteins after MYH1G-AS overexpression or knockdown. However, overexpression or interference of MYH1G-AS did not change the phosphorylation levels of the SMAD2 and SMAD3 proteins both in vitro and in vivo (Additional file 1: Fig. S13A, B). In view of MYH1G-AS reducing interaction of FGF18 to SMARCA5, which is a chromatin remodeler that can selectively mediate binding of distinct TFs [28], we further explored whether MYH1G-AS mediates the binding of TFs to the *SMAD4* promoter. First, promoter truncation experiments were performed and found that luciferase activities in the −558 ~ 0 region and −1444 ~ 0 region of the *SMAD4* promoter were promoted while luciferase activities in −994 ~ 0 region and −2000 ~ 0 region were restrained (Fig. 7L). Next, potential TF binding sites in transcriptional inhibitory regions of the *SMAD4* promoter were predicted by using gene-regulation (http://gene-regulation.com) and JASPAR software. The result showed that POU class 2 homeobox 1 (POU2F1) showed potential binding from the −1504 to −1493 region of the *SMAD4* promoter. Luciferase activities were increased with mutation of the POU2F1 binding site (Fig. 7M), suggesting that POU2F1 suppresses the transcription activity of *SMAD4*. The binding of POU2F1 to the *SMAD4* promoter was also verified by ChIP assay (Fig. 7N). *POU2F1* overexpression suppressed *SMAD4* expression, whereas *SMAD4* expression was upregulated after *POU2F1* interference (Fig. 7O, P). MYH1G-AS overexpression impeded the binding of POU2F1 to the *SMAD4* promoter and upregulated transcription activity of *SMAD4*, whereas the interaction of POU2F1 to the *SMAD4* promoter was promoted and *SMAD4* transcription activity was inhibited after MYH1G-AS interference (Fig. 7Q, R). On the contrary, *FGF18* or *SMRACR5* overexpression repressed the expression and transcription activity of *SMAD4* and hindered the binding of POU2F1 to the *SMAD4* promoter; *SMAD4* expression and transcription activity were facilitated and binding of POU2F1 was reinforced after *FGF18* or *SMRACR5* interference (Additional file 1: Fig. S13C-L), indicating that MYH1G-AS reduces the interaction of FGF18 to SMARCA5 to hinder the binding of POU2F1 to the *SMAD4* promoter, thus promoting the transcription activity and expression of *SMAD4*.

**Fig. 8:**
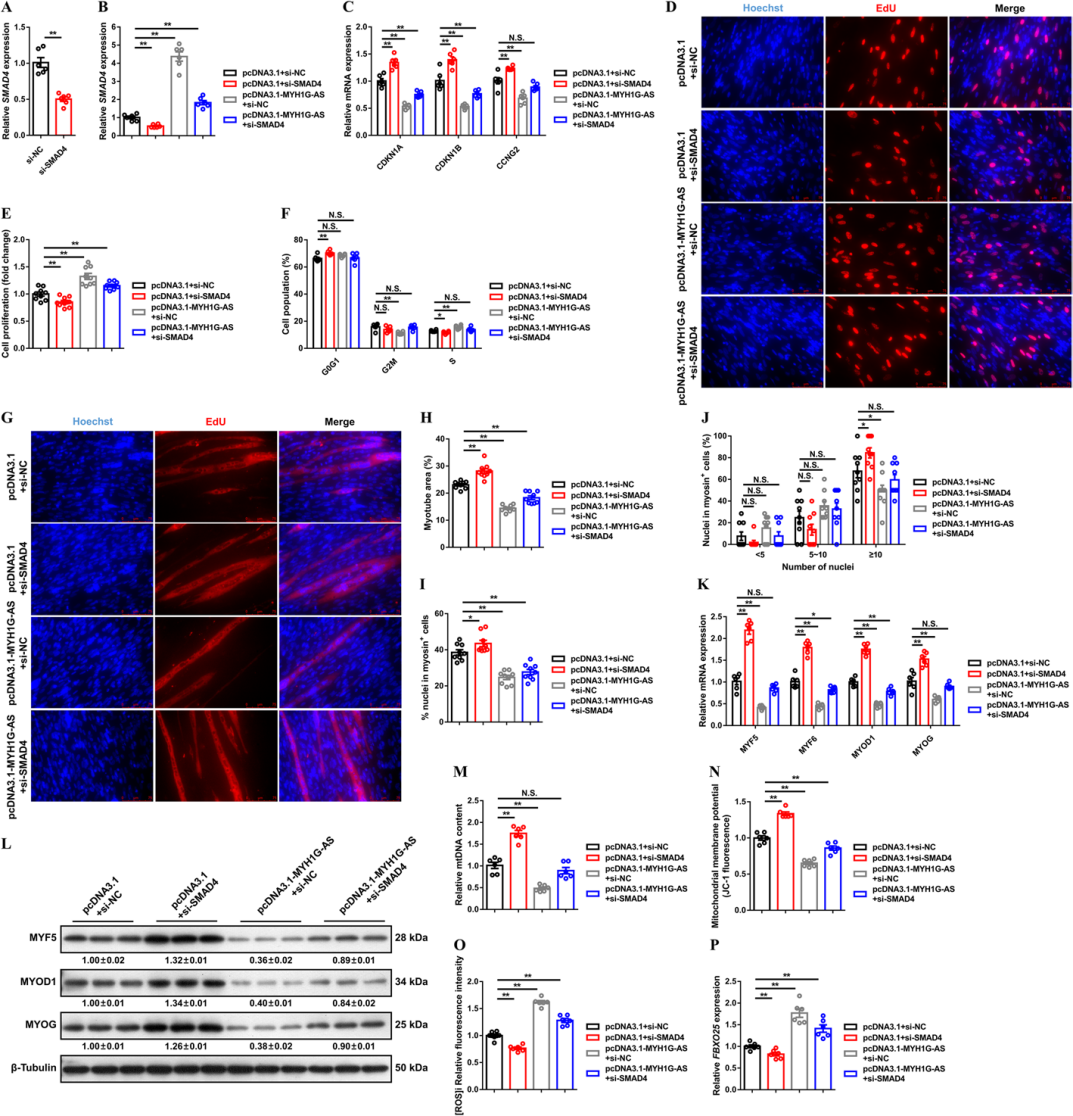
*SMAD4* is required for the function of MYH1G-AS. **A** Relative *SMAD4* expression after *SMAD4* interference. **B**–**P** Relative *SMAD4* expression (**B**), relative mRNA expression of several cell cycle-inhibiting genes (**C**), EdU proliferation assays (**D**), proliferation rate of myoblasts (**E**), cell cycle analysis (**F**), MyHC immunostaining (**G**), myotube area (**H**), differentiation index (**I**), myoblast fusion index (**J**), relative mRNA (**K**) and protein (**L**) expression levels of myoblast differentiation marker genes, relative mtDNA content (**M**), mitochondrial membrane potential (**N**), [ROS]i (**O**), and relative mRNA expressions of *FBXO25* (**P**) induced by the listed nucleic acids in CPMs. In panel **L**, the numbers shown below the bands are folds of band intensities relative to the control. Band intensities were quantified by ImageJ and normalized to β-tubulin. Results are presented as mean ± SEM. In panels **A**–**C**, **E**, **F**, **H**–**K**, and **M**–**P**, the statistical significance of differences between means was assessed using an independent sample *t*-test. (*P < 0.05; **P < 0.01; NS, no significant difference)

Specific siRNA against *SMAD4* was used to study whether *SMAD4* mediates the molecular function of MYH1G-AS (Fig. 8A). *SMAD4* interference cancelled out the promoting effect of MYH1G-AS on *SMAD4* expression (Fig. 8B). *SMAD4* inhibition suppressed myoblast proliferation but promoted myogenic differentiation, which neutralized the regulation of MYH1G-AS in myogenesis (Fig. 8C–L). In addition, *SMAD4* interference rescued the inhibition of mitochondria biogenesis and attenuated upregulated expression of *FBXO25*, which were induced by MYH1G-AS overexpression (Fig. 8M–P). Altogether, these results hinted that *SMAD4* is required for the function of MYH1G-AS.

## Discussion

Chromatin epigenome interaction, which functionally determines transcription and cell type, provides a powerful frame work for epigenetically classifying cellular substates [29]. During C2C12 differentiation, 385 DE lncRNAs, the transcription of which is determined by chromatin states around their transcriptional start sites, were found and identified as TF-lncRNA [30]. However, only the TF-lncRNA correlation network has been constructed, and the exact regulatory role has not been verified. To explore the genetic regulation during skeletal muscle development, we provide comprehensive insight into the transcriptome and chromatin accessibility in different myofibers. A total of 45 open chromatin-associated lncRNAs were found. Among them, MYH1G-AS, which is an antisense transcript of *MYH1G*, was found to be coordinately regulated by *SMAD3* and *SP2*.

As the most abundant RNA modification, m^6^A modification plays an important role in skeletal muscle development [19, 31]. However, *ALKBH5* is a well-known demethylase that has rarely been reported to regulate skeletal muscle development. In this study, *ALKBH5* was found to be highly expressed in fast-twitch myofibers. Moreover, *ALKBH5* modulated m^6^A demethylation of MYH1G-AS to maintain MYH1G-AS RNA stability, thus participating in the regulation of skeletal muscle development. Previous studies in mammals have found that multiple TFs can modulate *ALKBH5* transcription [32–34], but little is known in chicken. Here, we found *ALKBH5* transcription was repressed by *SP2*, indicated that *SP2* modulates m^6^A methylation to strengthen the suppression of MYH1G-AS expression.

Chromosome-associated RNA has been reported to be physically associated with chromatin and is widely involved in chromatin remodeling [10]. In this study, we found MYH1G-AS binds with FGF18 to inhibit FGF18 protein stabilization and reduce the interaction of FGF18 to SMARCA5, thus repressing chromatin accessibility and hindering the binding of POU2F1 to the *SMAD4* promoter. Notably, recent evidence has revealed that m^6^A modifications on chromatin-associated RNAs modulate chromatin accessibility and gene transcription [35, 36]. Here, we found that *ALKBH5*-mediated m^6^A demethylation enhances RNA stability of MYH1G-AS, which is a chromosome-associated lncRNA, and expands our understanding of the crosstalk between RNA modification and chromatin status.

It is well established that the TGF-β signaling pathway is widely involved in the regulation of cell growth, differentiation, and development [37–39]. As a family of signal transduction molecules, the SMAD family plays important roles in mediating the TGF-β signaling pathway [40–42]. *SMAD4* is the only member of the SMAD family with the common-mediator function. The receptor-regulated SMADs, such as SMAD2 and SMAD3, must combine with SMAD4 to form heterogenic complexes to exert their role in modulating the expression of their target genes [27]. Previous studies have reported cascading relationships among members of the SMAD family, but the expression regulation among members of the SMAD family remains poorly understood. In this study, we found MYH1G-AS regulated skeletal muscle development in vitro and in vivo through the *SMAD4*-dependent pathway. Given that MYH1G-AS, which is positively regulated by *SMAD3*, reduced interaction between FGF18 and SMARCA5 to remit the transcriptional inhibition of *SMAD4* by POU2F1, we concluded that *SMAD3* could promote *SMAD4* expression by mediating the transcription of MYH1G-AS. Our study presents a model for the expression regulation among members of the SMAD family and broadens insights into the interaction of cascade molecules in the TGF-β signaling pathway.

## Conclusions

In summary, we identified a chromatin-associated lncRNA that regulates skeletal muscle development. Our results reveal a new pattern of the regulation of lncRNA expression at diverse levels and help expound the regulation of m^6^A methylation on chromatin status.

### Supplementary Information

**Additional file 1: Table S1.** Information of primers. **Table S2**. Sequences of potential ORFs of MYH1G-AS. **Table S3.** Oligonucleotide sequences in this study. **Fig. S1. **Relative β-Tubulin and GAPDH protein expression in myoblast proliferation (myoblast cultured in growth medium [GM]) and differentiation (myoblast cultured in differentiation medium from 1 to 5 day [DM1 to DM5; DM indicate differentiation day]) periods. The numbers shown below the bands were folds of band intensities relative to control. Band intensities were quantified by ImageJ and normalized to β-Tubulin. Data are expressed as a fold-change relative to the control. Results are presented as mean ± SEM. **Fig. S2. **GO functions and KEGG pathways analysis of differentially expressed genes and ATAC-seq peaks between pectoralis major and soleus in 7-week-old Xinghua chicken. (**A**, **B**) GO functions (**A**) and KEGG pathways (**B**) analysis of differentially expressed genes between pectoralis major (PEM) and soleus (SOL) in 7-week-old Xinghua chicken. (**C**-**D**) GO functions (**C**) and KEGG pathways (**D**) analysis of differentially expressed ATAC-seq peaks between PEM and SOL in 7-week-old Xinghua chicken. **Fig. S3. **Heatmap of ATAC-seq signals at transcriptional start site. (**A**) Heatmap of ATAC-seq signals at transcriptional start site (TSS) in PEM samples. (**B**) Heatmap of ATAC-seq signals at TSS in SOL samples. **Fig. S4. **Characterization of MYH1G-AS. (**A**) Results of MYH1G-AS 3’ RACE and 5’ RACE. (**B**) The full-length sequence of MYH1G-AS. Coordinates are listed according to bGalGal1.mat.broiler.GRCg7b reference Annotation Release 106. (**C**) Conservative analysis of MYH1G-AS performed by using the NCBI’s BLAST. A total of eighteen species, including *Anas platyrhynchos*, *Anser cygnoides*, *Apteryx mantelli mantelli*, *Aquila chrysaetos*, *Bos taurus*, *Coturnix japonica*, *Gallus gallus*,*Geospiza fortis*, *Homo sapiens*, *Meleagris gallopavo*, *Melopsittacus undulatus*, *Mus musculus*, *Numida meleagris*, *Ovis aries*,*Pan troglodytes*, *Rattus norvegicus*, *Sus scrofa* and *Zebra finch* were used for Nucleotide BLAST. Top 5 most conservative results were listed above. **Fig. S5. **Prediction and identification of potential m^6^A modification sites on MYH1G-AS. (**A**) Potential m^6^A modification sites on MYH1G-AS were predict by using the SRAMP (http://www.cuilab.cn/sramp) software. (**B**–**G**) Relative single-base elongation and ligation-based PCR amplification method (SELECT) product at 250 (**B**), 436 (**C**), 495 (**D**), 970 (**E**), 1042 (**F**), and 1116 (**G**) sites of MYH1G-AS after *ALKBH5* overexpression or interference. (**H**–**M**) Relative SELECT product at 250 (**H**), 436 (**I**), 495 (**J**), 970 (**K**), 1042 (**L**), and 1116 (**M**) sites of MYH1G-AS after *ALKBH5* overexpression or interference. Results are presented as mean ± SEM. In panels **B**-**M**, the statistical significance of differences between means was assessed using an independent sample *t*-test. (NS, no significant difference). **Fig. S6.**GO functions and KEGG pathways analysis of differentially expressed genes between control group and MYH1G-AS interference. (**A**)GO functions analysis of differentially expressed genes between control group and MYH1G-AS interference. (**B**) KEGG pathways analysis of differentially expressed genes between control group and MYH1G-AS interference. **Fig. S7. **Overexpression of MYH1G-AS promotes myoblast proliferation but inhibits myoblast differentiation. (**A**–**L**) Relative MYH1G-AS expression (**A**), relative mRNA expressions of several cell cycle-inhibiting genes (**B**), EdU proliferation assays (**C**), proliferation rate of myoblasts (**D**), cell cycle analysis €, CCK-8 assays (**F**), MyHC immunostaining (**G**), myotube area (**H**), differentiation index (**I**), myoblast fusion index (**J**), and relative mRNA (**K**) and protein (**L**) expression levels of myoblast differentiation marker genes with MYH1G-AS overexpressionin vitro. In panel **L**, the numbers shown below the bands were folds of band intensities relative to control. Band intensities were quantified by ImageJ and normalized to β-Tubulin. Data are expressed as a fold-change relative to the control. Results are presented as mean ± SEM. In panels **A**, **B**, **D**–**F**, and **H**–**K**, statistical significance of differences between means was assessed using independent sample *t*-test. (* *P* < 0.05; ** *P* < 0.01). **Fig. S8. **Overexpression of MYH1G-AS represses mitochondria biogenesis to drive the transformation of slow-twitch to fast-twitch myofiber and induces muscle atrophy. (**A**-**Q**) Relative MYH1G-AS expression (**A**), relative mtDNA content (**B**), mitochondrial membrane potential (**C**), intracellular ROS ([ROS]i) (**D**), relative glycogen content (**E**), immunohistochemistry analysis of MYH1A/MYH7B (**F**), MYH1A/MYH7B protein content (**G**), frequency distribution of MYH1A^+^ (**H**) and MYH7B^+^ (**I**) myofiber CSA,  relative protein expression of MYH1A and MYH7B (**J**), relative mRNA expression of several fast- and slow-twitch myofiber genes (**K**), relative enzymes activity of LDH and SDH (**L**), relative gastrocnemius muscle weight (**M**), H&E staining (**N**), frequency distribution of fiber CSA (**O**), and relative mRNA (**P**) and protein (**Q**) expression of* FBXO25* in gastrocnemius with MYH1G-AS overexpression. In panel **J** and **Q**, the numbers shown below the bands were folds of band intensities relative to control. Band intensities were quantified by ImageJ and normalized to β-Tubulin. Data are expressed as a fold-change relative to the control. Results are showed as mean ± SEM. In panels **A**–**E**,** G**, **K**–**M**, and **P**, statistical significance of differences between means was assessed using independent sample *t*-test. (* *P* < 0.05; ** *P* < 0.01). **Fig. S9.** The mRNA level of* MYH1G* and *FGF18* didn’t change with MYH1G-AS overexpression and knockdown both in vitro and in vivo. (**A**-**B**) Relative mRNA expression of *MYH1G* with MYH1G-AS overexpression and knockdown in vitro (**A**) and in vivo (**B**). (**C**-**D**)Relative mRNA expression of *FGF18* with MYH1G-AS overexpression and knockdown in vitro (**C**) and in vivo (**D**). Results are shown as mean ± SEM. In all panels, statistical significance of differences between means was assessed using independent sample* t*-test. (NS, no significant difference). **Fig. S10.** The location and expression analysis of *FGF18*.(**A**) Subcellular location of FGF18 protein annotated by UniProt Knowledgebase (https://www.uniprot.org/). (**B**) Tissue expression profiles of *FGF18*. The horizontal axis and vertical axis indicate different tissues and their relative expression values, respectively. (**C**) Relative *FGF18* expression during CPM proliferation and differentiation. Results are presented as mean ± SEM. In panels **B**-**C**, statistical significance of differences between means was assessed using independent sample *t*-test. (* *P* < 0.05; ** *P* < 0.01). **Fig. S11. ***FGF18* inhibits myoblast proliferation, promotes myoblast differentiation, and facilitates mitochondria biogenesis. (**A**-**R**) Relative mRNA (**A**) and protein **(B**) expression levels of *FGF18*, relative mRNA expressions of several cell cycle-inhibiting genes (**C**), EdU proliferation assays (**D**), proliferation rate of myoblasts (**E**), cell cycle analysis (**F**), CCK-8 assays (**G**-**H**), MyHC immunostaining (**I**), myotube area (**J**), differentiation index (**K**), myoblast fusion index (**L**), relative mRNA (**M**) and protein (**N**) expression levels of myoblast differentiation marker genes, relative mtDNA content (**O**), mitochondrial membrane potential (**P**), intracellular ROS ([ROS]i) (**Q**), and relative *FBXO25* expression (**R**) with *FGF18* overexpression or interference in vitro. In panels **B** and **N**, the numbers shown below the bands were folds of band intensities relative to control. Band intensities were quantified by ImageJ and normalized to β-Tubulin. Data are expressed as a fold-change relative to the control. Results are showed as mean ± SEM. In panels **A**, **C**,** E**-**H**, **J**-**M**, and **O**-**R**, statistical significance of differences between means was assessed using independent sample* t*-test. (* *P* < 0.05; ** *P* < 0.01). **Fig. S12.** Neither MYH1G-AS nor *FGF18* regulate the mRNA and protein expression of *SMARCA5*. (**A**-**D**) Relative mRNA (**A** and **C**) and protein (**B** and **D**) expression levels of *SMARCA5* after MYH1G-AS overexpression or interference in vitro (**A**-**B**) orin vivo (**C**-**D**). (**E**-**F**) Relative mRNA (**E**) and protein (**F**) expression levels of *SMARCA5* after *FGF18* overexpression or interference. In panels **B**, **D**, and **F**, the numbers shown below the bands were folds of band intensities relative to control. Band intensities were quantified by ImageJ and normalized to β-Tubulin. Data are expressed as a fold-change relative to the control. Results are presented as mean ± SEM. In panels **A**, **C**,and **E**, statistical significance of differences between means was assessed using independent sample* t*-test. (NS, no significant difference). **Fig. S13.** *FGF18* and *SMARCA5* promotes the expression and transcription of *SMAD4*. (**A**-**B**) Protein expression levels of phosphorylated SMAD2 and phosphorylated SMAD3 after MYH1G-AS overexpression or interference in vitro (**A**) or in vivo (**B**). (**C**-**F**) Relative *SMAD4 *mRNA expression (**C**), protein expression levels of *SMAD4*, phosphorylated SMAD2 and phosphorylated SMAD3 (**D**), the transcriptional activity of *SMAD4* core promoter region (**E**), and ChIP analysis of the binding capacity of POU2F1 to *SMAD4* promoter (**F**) with *FGF18* overexpression or knockdown. (**G**-**L**) Relative mRNA (**G**) and protein (**H**) expression levels of *SMARCA5*, relative *SMAD4 *mRNA expression (**I**), protein expression levels of *SMAD4*, phosphorylated SMAD2 and phosphorylated SMAD3 (**J**), the transcriptional activity of *SMAD4* core promoter region (**K**), and ChIP analysis of the binding capacity of POU2F1 to *SMAD4* promoter (**L**) with *SMARCA5* overexpression or knockdown. In panels **A**-**B**, **D**, **H**, and **J**, the numbers shown below the bands were folds of band intensities relative to control. Band intensities were quantified by ImageJ and normalized to β-Tubulin. Data are expressed as a fold-change relative to the control. Results are presented as mean ± SEM. In panels **C**, **E**-**G**, **I**, and **K**-**L**, statistical significance of differences between means was assessed using independent sample* t*-test. (* *P* < 0.05; ** *P*< 0.01).

** Additional file 2. **Raw images of Western blot.

**Additional file 3: Table S4. **Differential expression analysis of genes between pectoralis major and soleus in 7-week-old Xinghua chicken.

** Additional file 4: Table S5.** Differential expression analysis of lncRNAs between pectoralis major and soleus in 7-week-old Xinghua chicken.

** Additional file 5: Table S6. **Differential analysis of ATAC-seq peaks between pectoralis major and soleus in 7-week-old Xinghua chicken.

** Additional file 6: Table S7.** List of open chromatin-associated lncRNAs.

**Additional file 7: Table S8. **Differential expression analysis of genes between control group and MYH1G-AS interference in CPMs.

** Additional file 8: Table S9.** Comparative metabolome analysis of control group versus lncRNA MYH1G-AS knockdown gastrocnemius.

** Additional file 9: Table S10.** lncRNA MYH1G-AS specific binding proteins identified by RNA pull-down coupled to mass spectrometry.

** Additional file 10: Table S11.** FGF18 specific interacting proteins identified by Co-IP coupled to mass spectrometry.

## Data Availability

The datasets used and/or analyzed during the current study are available from the corresponding author on reasonable request.

## Abbreviations

ASO: Antisense oligonucleotide
ATAC-seq: Assay for transposase-accessible chromatin with high-throughput sequencing
CCK-8: Cell counting kit-8
ChIP: Chromatin immunoprecipitation
CHX: Cycloheximide
CPM: Chicken primary myoblast
CSA: Cross-sectional area
DE: Differentially expressed
EdU: 5-ethynyl-2′-deoxyuridine
FGF18: Fibroblast growth factor 18
FISH: RNA fluorescence in situ hybridization
HE: Hematoxylin and eosin
IF: Immunofluorescence
IHC: Immunohistochemistry
KEGG: Kyoto Encyclopedia of Genes and Genomes
LDH: Lactic dehydrogenase
lncRNA: Long noncoding RNA
m^6^A: *N*^6^-methyladenosine
mtDNA: Mitochondrial DNA
ncRNA: Noncoding RNA
ORF: Open Reading Frame
PEM: Pectoralis major
POU2F1: POU class 2 homeobox 1
RACE: Rapid amplification of complementary DNA ends
RIP: RNA immunoprecipitation
RNA-seq: RNA sequencing
ROS: Reactive oxygen species
RT-qPCR: Real-time quantitative polymerase chain reaction
SDH: Succinate dehydrogenase
SELECT: Single-base elongation and ligation-based qPCR amplification method
SMAD4: SMAD family member 4
SMARCA5: SWI/SNF-related matrix-associated actin-dependent regulator of chromatin subfamily A member 5
SOL: Soleus
TF: Transcription factor
TSS: Transcriptional start site
UPS: Ubiquitin-proteasome system
