# Supplementary material for: MYH1G-AS is a chromatin-associated lncRNA that regulates skeletal muscle development in chicken
Source: Cell Mol Biol Lett. 2024 Jan 4;29:9. doi: 10.1186/s11658-023-00525-x (PMC10765903; doi:10.1186/s11658-023-00525-x)
Supplement: Supplementary file 1 — Additional file 1: Table S1. Information of primers. Table S2. Sequences of potential ORFs of MYH1G-AS. Table S3. Oligonucleotide sequences in this study. Fig. S1. Relative β-Tubulin and GAPDH protein expression in myoblast proliferation (myoblast cultured in growth medium [GM]) and differentiation (myoblast cultured in differentiation medium from 1 to 5 day [DM1 to DM5; DM indicate differentiation day]) periods. The numbers shown below the bands were folds of band intensities relative to control. Band intensities were quantified by ImageJ and normalized to β-Tubulin. Data are expressed as a fold-change relative to the control. Results are presented as mean ± SEM. Fig. S2. GO functions and KEGG pathways analysis of differentially expressed genes and ATAC-seq peaks between pectoralis major and soleus in 7-week-old Xinghua chicken. (A, B) GO functions (A) and KEGG pathways (B) analysis of differentially expressed genes between pectoralis major (PEM) and soleus (SOL) in 7-week-old Xinghua chicken. (C-D) GO functions (C) and KEGG pathways (D) analysis of differentially expressed ATAC-seq peaks between PEM and SOL in 7-week-old Xinghua chicken. Fig. S3. Heatmap of ATAC-seq signals at transcriptional start site. (A) Heatmap of ATAC-seq signals at transcriptional start site (TSS) in PEM samples. (B) Heatmap of ATAC-seq signals at TSS in SOL samples. Fig. S4. Characterization of MYH1G-AS. (A) Results of MYH1G-AS 3’ RACE and 5’ RACE. (B) The full-length sequence of MYH1G-AS. Coordinates are listed according to bGalGal1.mat.broiler.GRCg7b reference Annotation Release 106. (C) Conservative analysis of MYH1G-AS performed by using the NCBI’s BLAST. A total of eighteen species, including Anas platyrhynchos, Anser cygnoides, Apteryx mantelli mantelli, Aquila chrysaetos, Bos taurus, Coturnix japonica, Gallus gallus,Geospiza fortis, Homo sapiens, Meleagris gallopavo, Melopsittacus undulatus, Mus musculus, Numida meleagris, Ovis aries,Pan troglodytes, Rattus norvegicus, Sus scrofa an [file 11658_2023_525_MOESM1_ESM.docx]

**Table S1.** Information of Primers.

| **Primer name** | **Primer sequences (5’ to 3’)** | **Usage** |
| --- | --- | --- |
| qPCR-MYH1G-AS | F: CATTCTGCCAAGTCCTTC | qPCR |
|  | R: CTGACCAAACAGGGAGTG |  |
| qPCR-SMAD3 | F: CCACCGTTGGACGATTACA | qPCR |
|  | R: TCACTCAAATAGCCTGGAGGA |  |
| qPCR-SP2 | F: AAAAGCGACGACGGATGGC | qPCR |
|  | R: TTCCCGCAGAAGACCCAGT |  |
| qPCR-ALKBH5 | F: CATCGTCTCCGTGTCGTTCT | qPCR |
|  | R: GTGTAATTTCGTCGGCTGCG |  |
| qPCR-CDKN1A | F: CCCGTAGACCACGAGCAGAT | qPCR |
|  | R: CGTCTCGGTCTCGAAGTTGA |  |
| qPCR-CDKN1B | F: TCGCTGTGCTGGGCTGAA | qPCR |
|  | R: CAAGGACGAAAGGATGTGGG |  |
| qPCR-CCNG2 | F: TGCCAACAATACCAGAGG | qPCR |
|  | R: TACAGAATACCACAATCCC |  |
| qPCR-MYF5 | F: GCTCTTGAGGGAACAGGTGGA | qPCR |
|  | R: TAGCCGTGGGGCATCTCG |  |
| qPCR-MYF6 | F: CGCCATCAGCTACATCGAGAG | qPCR |
|  | R: CCGCAGGTGCTCAGGAAGT |  |
| qPCR-MYOD1 | F: GCTACTACACGGAATCACCAAAT | qPCR |
|  | R: CTGGGCTCCACTGTCACTCA |  |
| qPCR-MYOG | F: CGGAGGCTGAAGAAGGTGAA | qPCR |
|  | R: CGGTCCTCTGCCTGGTCAT |  |
| qPCR-COX2 | F: GTAGATGCCCAAGAAGTT | qPCR |
|  | R: GTTTGATTTAGTCGTCCAG |  |
| qPCR-β-globin | F: CAGCCAGGTGGAGGATTT | qPCR |
|  | R: GAATAGGAGGACCCTCTGTTAG |  |
| qPCR-FBXO25 | F: TAGAAACTATCCGCCTGTG | qPCR |
|  | R: CCCAATGTAATAATGTCCC |  |
| qPCR-MYH1G | F: AAGAACTAAAGCGGCAACTA | qPCR |
|  | R: GCTGAATAGCATCCGTCTC |  |
| qPCR-FGF18 | F: AGGGAGAAGACGGAGATAA | qPCR |
|  | R: TCTCAATGAAGACGCACTC |  |
| qPCR-SMARCA5 | F: GCACTCGGTTTGAAGAATC | qPCR |
|  | R: AGACCCATTTCATCTGCTAG |  |
| qPCR-SMAD4 | F: CCAGCAAGTGCGTCACCATC | qPCR |
|  | R: GCGAACTGGCAGAACTTGGAG |  |
| qPCR-POU2F1 | F: TGATGTTGGGCTCGCTAT | qPCR |
|  | R: TTCCTCCTACGGTTCACG |  |
| qPCR-GAPDH | F: GGAGAAACCAGCCAAGTA | qPCR |
|  | R: GCATCAAAGGTGGAGGAAT |  |
| qPCR-U6 | F: CTCGCTTCGGCAGCACA | qPCR |
|  | R: AACGCTTCACGAATTTGCGT |  |
| qPCR-TNNC1 | F: GTTGAGCAGTTGACAGAAGA | qPCR |
|  | R: GAACCATCATAACAAGGAAC |  |
| qPCR-TNNC2 | F: GAGCAGCAAAGATGGCGTCA | qPCR |
|  | R: ATCACCGTGCCCAACTCCTT |  |
| qPCR-TNNI1 | F: GAGGAGTGGGAGCAGGAGAT | qPCR |
|  | R: TTCGTCCACAATCTCAACCT |  |
| qPCR-TNNT1 | F: GAGCCGCACGGAGAAGGAGC | qPCR |
|  | R: CCCGAAGTGGGGCATGTTGG |  |
| qPCR-TNNT3 | F: AGAGGGAAGAAGCAAACAGC | qPCR |
|  | R: GTCCCACAGTTCCTTAGCCT |  |
| qPCR-β-actin | F: GATATTGCTGCGCTCGTTG | qPCR |
|  | R: TTCAGGGTCAGGATACCTCTTT |  |
| 5' RACE-MYH1G-AS | F: AAGCAGTGGTATCAACGCAGAGT | RACE |
|  | R: ACAGTAGGCAGGTGGTGGGGTGCTCA |  |
| 3' RACE-MYH1G-AS | F: GGAGGCAGGGAGATACAAACACAGCCAT | RACE |
|  | R: ACTCTGCGTTGATACCACTGCTT |  |
| MYH1G-AS-ORF1-FLAG | F: **GGATCC**ATGAATGCACGAATGTTGT | Vector construction |
|  | R: **TCTAGA**GAATTTGCAGGAAAGGATTTC |  |
| MYH1G-AS-ORF2-FLAG | F: **GGATCC**ATGCGGTTCTCCAGCAGA | Vector construction |
|  | R: **TCTAGA**CTGCAGCAGTAGACTTGTCAGG |  |
| MYH1G-AS-ORF3-FLAG | F: **GGATCC**ATGAAGAGCATTCCAATGG | Vector construction |
|  | R: **TCTAGA**CAGTAGGCAGGTGGTGGG |  |
| MYH1G-AS-ORF4-FLAG | F: **GGATCC**ATGGGAATTTCCTCGTCT | Vector construction |
|  | R: **TCTAGA**CAGTAGGCAGGTGGTGG |  |
| MYH1G-AS-ORF5-FLAG | F: **GGATCC**ATGAGCACCCCACCACCT | Vector construction |
|  | R: **TCTAGA**TTCATGCTTTTGGGTGGGA |  |
| MYH1G-AS-ORF6-FLAG | F: **GGATCC**ATGTGCAGGTGTCTTGAAA | Vector construction |
|  | R: **TCTAGA**CAGCATTCCTCTGCCTCC |  |
| pGL3-MYH1G-AS-WT | F: **GCTAGC**CCAGCATGCAACCACTGC | Vector construction |
|  | R: **AAGCTT**TAAATGCCCTTTTGATATGGA |  |
| pGL3-MYH1G-AS-SMAD3-MT | F: TGTTATTGTAGGGTCGTGAGCTCACTCAGTTTGCTCTGTA | Vector construction |
|  | R: TACAGAGCAAACTGAGTGAGCTCACGACCCTACAATAACA |  |
| pGL3-MYH1G-AS-SP2-MT | F: CCACTGACAGAGAATTAAAACAAGAGTATTTAGCAACATTTCTGC | Vector construction |
|  | R: GCAGAAATGTTGCTAAATACTCTTGTTTTAATTCTCTGTCAGTGG |  |
| pcDNA3.1-SMAD3 | F: **GCTAGC**ATGTCCTCCATCCTGCCGT | Vector construction |
|  | R: **TCTAGA**TTAGGAGACGCTGGAGCAGC |  |
| pcDNA3.1-SP2-FLAG | F: **GCTAGC**ATGGTCAACATCCTCGCCG | Vector construction |
|  | R: **TCTAGA**CTACAAGTTCTTGGTGATCAGGTGG |  |
| pcDNA3.1-SP2 | F: **GCTAGC**ATGGTCAACATCCTCGCCG | Vector construction |
|  | R: **TCTAGA**CTACTTGTCATCGTCGTCCTTGTAATCCAAGTTCTTGGTGATCAGGTGG |  |
| pcDNA3.1-ALKBH5 | F: **GCTAGC**ATGGCCGGCAGCGGCTAC | Vector construction |
|  | R: **CTCGAG**CAGTGTCGCCTCATTTTAACTTT |  |
| pGL3-ALKBH5-(-450~0) | F: **GCTAGC**GGGCCAGGCTGTATTTGCG | Vector construction |
|  | R: **AAGCTT**CCCACCGCGCCGGCACTGCT |  |
| pGL3-ALKBH5-(-940~0) | F: **GCTAGC**CGAGGTGCGGACCCTTTGGA | Vector construction |
|  | R: **AAGCTT**CCCACCGCGCCGGCACTGCT |  |
| pGL3-ALKBH5-(-1428~0) | F: **GCTAGC**GCAGCAAGCACAGGCACTCA | Vector construction |
|  | R: **AAGCTT**CCCACCGCGCCGGCACTGCT |  |
| pGL3-ALKBH5-(-2000~0) | F: **GCTAGC**GCCGGGGCTGCACAGGG | Vector construction |
|  | R: **AAGCTT**CCCACCGCGCCGGCACTGCT |  |
| pGL3-ALKBH5-(-940~-451)-WT | F: **GCTAGC**CGAGGTGCGGACCCTTT | Vector construction |
|  | R: **AAGCTT**CGCGTCAAGGGCTTGTATAA' |  |
| pGL3-ALKBH5-(-890~-876)-MT | F: GGGACCGCCCTCCCAAAAAATAAAAAATAACCCAGGAGCCCGTGG | Vector construction |
|  | R: CCACGGGCTCCTGGGTTATTTTTTATTTTTTGGGAGGGCGGTCCC |  |
| pGL3-ALKBH5-(-508~-494)-MT | F: CCCCAACGTGACCCCAGAAAGAAAAAAGAATCGCTGTAACCCCGC | Vector construction |
|  | R: GCGGGGTTACAGCGATTCTTTTTTCTTTCTGGGGTCACGTTGGGG |  |
| pGL3-ALKBH5-(-896~-882)-MT | F: GACCCCGGGACCGCCAGAAACAAAAATAAACCCGCCCCCAGGAGC | Vector construction |
|  | R: GCTCCTGGGGGCGGGTTTATTTTTGTTTCTGGCGGTCCCGGGGTC |  |
| pGL3-ALKBH5-(-512~-498)-MT | F: CTGTCCCCAACGTGAAAAAAGAAAGAAAAACTCCTCGCTGTAACC | Vector construction |
|  | R: GGTTACAGCGAGGAGTTTTTCTTTCTTTTTTCACGTTGGGGACAG |  |
| pGL3-ALKBH5-(-883~-869)-MT | F: CCCTCCCACCCCCGCAAAAATAAAAACTTCGCCCGTGGCCAAGTT | Vector construction |
|  | R: AACTTGGCCACGGGCGAAGTTTTTATTTTTGCGGGGGTGGGAGGG |  |
| pcDNA3.1-MYH1G-AS | F: **GCTAGC**GACAAAACCTTTCATCCCTA | Vector construction |
|  | R: **TCTAGA**TTCAGTTTGCCAGACAATG |  |
| pLVX-mCMV-ZsGreen-IRES-Puro-MYH1G-AS | F: **ACTAGT**GACAAAACCTTTCATCCCTACGCTT | Vector construction |
|  | R: **GGATCC**TTCAGTTTGCCAGACAATGCTTTAC |  |
| pcDNA3.1-FGF18-FLAG | F: **GCTAGC**ATGTATTCACTGCTCTCCGCCT | Vector construction |
|  | R: **TCTAGA**TTAACTGGGGTTGGTGGGTC |  |
| pcDNA3.1-FGF18 | F: **GCTAGC**ATGTATTCACTGCTCTCCGCCT | Vector construction |
|  | R: **TCTAGA**TTACTTGTCATCGTCGTCCTTGTAATCACTGGGGTTGGTGGGTC |  |
| pcDNA3.1-SMARCA5-Myc | F: **GCTAGC**ATGTCCGCCGGGCAGCA | Vector construction |
|  | R: **CTCGAG**TCACAGATCCTCTTCAGAGATGAGTTTCTGCTCCAGCTTTAGCTTCTTTTTTC |  |
| pcDNA3.1-SMARCA5 | F: **GCTAGC**ATGTCCGCCGGGCAGCA | Vector construction |
|  | R: **CTCGAG**TCACAGCTTTAGCTTCTTTTTTC |  |
| pGL3-SMAD4-(-558~0) | F: **GCTAG**CTCCGCCTCCGTGCCGTTCCTTA | Vector construction |
|  | R: **CTCGAG**GCATCGCCGCCCGCCCC |  |
| pGL3-SMAD4-(-944~0) | F: **GCTAGC**CCCCGAAACGAACGACTGAAGG | Vector construction |
|  | R: **CTCGAG**GCATCGCCGCCCGCCCC |  |
| pGL3-SMAD4-(-1444~0) | F: **GCTAGC**CCAAAGCCCGAGATCCCACCCACA | Vector construction |
|  | R: **CTCGAG**GCATCGCCGCCCGCCCC |  |
| pGL3-SMAD4-(-2000~0) | F: **GCTAGC**ATGGCCCCCAGGATGG | Vector construction |
|  | R: **CTCGAG**GCATCGCCGCCCGCCCC |  |
| pGL3-SMAD4-(-2000~-1445) | F: **GCTAGC**ATGGCCCCCAGGATGG | Vector construction |
|  | R: **CTCGAG**GATGCCATCTGCACATTG |  |
| pGL3-SMAD4-(-1506~-1491)-MT | F: AGGTGCTTCCCCATATTCTGGGTACGTGCCTGCCTCCTAAAAGCA | Vector construction |
|  | R: TGCTTTTAGGAGGCAGGCACGTACCCAGAATATGGGGAAGCACCT |  |
| pcDNA3.1-POU2F1-FLAG | F: **GCTAGC**ATGAACAATCCGTCAGAAAC | Vector construction |
|  | R: **TCTAGA**TCACTTGTCATCGTCGTCCTTGTAATCCTGTGCCTTGGAGGCAGT |  |
| pcDNA3.1-POU2F1 | F: **GCTAGC**ATGAACAATCCGTCAGAAAC | Vector construction |
|  | R: **TCTAGA**TCACTGTGCCTTGGAGGCAGT |  |
| ChIP-MYH1G-AS-SMAD3 | F: GCCATGCTGCTGTTATTG | ChIP-qPCR |
|  | R: AGCGTAGGGATGAAAGGT |  |
| ChIP-MYH1G-AS-SP2 | F: CCTCTGCGGTAGCAACAT | ChIP-qPCR |
|  | R: GAAGCGTAGGGATGAAAGG |  |
| ChIP-ALKBH5-SP2 | F: AGGTGCGGACCCTTTGGA | ChIP-qPCR |
|  | R: TGATCTCCACGTACGGAGAGC |  |
| ChIP-SMAD4-POU2F1 | F: GTAAAGCAGGGCTGGGAT | ChIP-qPCR |
|  | R: GCTTTTAGGAGGCATTACA |  |
| MYH1G-AS-250 X-site | Up: TAGCCAGTACCGTAGTGCGTGCAACAGAGTCCTTTTAGAGAG | SELECT |
|  | Down: CTGTCTTCTGCATCCAGTACCAGAGGCTGAGTCGCTGCAT |  |
| MYH1G-AS-250 N-site | Up: TAGCCAGTACCGTAGTGCGTGAGTCCTTTTAGAGAGTCTGTC | SELECT |
|  | Down: TCTGCATCCAGTACAACATTCAGAGGCTGAGTCGCTGCAT |  |
| MYH1G-AS-263 X-site | Up: TAGCCAGTACCGTAGTGCGTGGGAAAGGATTTCCCAACAGAG | SELECT |
|  | Down: CCTTTTAGAGAGTCTGTCTTCAGAGGCTGAGTCGCTGCAT |  |
| MYH1G-AS-263 N-site | Up: TAGCCAGTACCGTAGTGCGTGGATTTCCCAACAGAGTCCTTT | SELECT |
|  | Down: AGAGAGTCTGTCTTCTGCATCAGAGGCTGAGTCGCTGCAT |  |
| MYH1G-AS-436 X-site | Up: TAGCCAGTACCGTAGTGCGTGAGAATGTGGGAAGTACCACAG | SELECT |
|  | Down: CACATCCACAGACAACCTTCCAGAGGCTGAGTCGCTGCAT |  |
| MYH1G-AS-436 N-site | Up: TAGCCAGTACCGTAGTGCGTGTGGGAAGTACCACAGTCACAT | SELECT |
|  | Down: CACAGACAACCTTCAGTATCCAGAGGCTGAGTCGCTGCAT |  |
| MYH1G-AS-495 X-site | Up: TAGCCAGTACCGTAGTGCGTGGCTGGAGAACCGCATCTCCAG | SELECT |
|  | Down: TTTCCCAGTGATGACTCCTCCAGAGGCTGAGTCGCTGCAT |  |
| MYH1G-AS-495 N-site | Up: TAGCCAGTACCGTAGTGCGTGGAACCGCATCTCCAGTTTTCC | SELECT |
|  | Down: AGTGATGACTCCTCTTGCCGCAGAGGCTGAGTCGCTGCAT |  |
| MYH1G-AS-970 X-site | Up: TAGCCAGTACCGTAGTGCGTGGGCTCAATTCCTCAGCTCGAG | SELECT |
|  | Down: TTTTAATCTGCTTTCAAGACCAGAGGCTGAGTCGCTGCAT |  |
| MYH1G-AS-970 N-site | Up: TAGCCAGTACCGTAGTGCGTGATTCCTCAGCTCGAGTTTTTA | SELECT |
|  | Down: TCTGCTTTCAAGACACCTGCCAGAGGCTGAGTCGCTGCAT |  |
| MYH1G-AS-1042 X-site | Up: TAGCCAGTACCGTAGTGCGTGGGATCTGCAGCCGTGCAGGAG | SELECT |
|  | Down: CTTTCTGTGTTACAGCATTCCAGAGGCTGAGTCGCTGCAT |  |
| MYH1G-AS-1042 N-site | Up: TAGCCAGTACCGTAGTGCGTGGCAGCCGTGCAGGAGTCTTTC | SELECT |
|  | Down: GTGTTACAGCATTCCTCTGCCAGAGGCTGAGTCGCTGCAT |  |
| MYH1G-AS-1116 X-site | Up: TAGCCAGTACCGTAGTGCGTGCAGATGAGATCGTATAAAATG | SELECT |
|  | Down: CCCTGCAGCAAGACTGCCCTCAGAGGCTGAGTCGCTGCAT |  |
| MYH1G-AS-1116 N-site | Up: TAGCCAGTACCGTAGTGCGTGAGATCGTATAAAATGTCCCTG | SELECT |
|  | Down: AGCAAGACTGCCCTGTCTCCCAGAGGCTGAGTCGCTGCAT |  |
| SELECT qPCR | Up: ATGCAGCGACTCAGCCTCTG | SELECT |
|  | Down: TAGCCAGTACCGTAGTGCGTG |  |

Sequences in bold represent the enzyme cutting sites.

**Table S2**. Sequences of potential ORFs of *MYH1G-AS*.

**ORF1:** ATGAATGCACGAATGTTGTACTGGATGCAGAAGACAGACTCTCTAAAAGGACTCTGTTGGGAAATCCTTTCCTGCAAATTCTGA

**ORF2:** ATGCGGTTCTCCAGCAGAGAGGAAACAAATGCACCTTTCACAACAGAGTTCCCTGAGTACCTGACAGATCTACTGCTGCAGTGA

**ORF3:** ATGAAGAGCATTCCAATGGGAATTTCCTCGTCTGCCATTGGAGGCAGGGAGATACAAACACAGCCATTTGAAAACCTTGCCAGCCATGAGCACCCCACCACCTGCCTACTGTAA

**ORF4:** ATGGGAATTTCCTCGTCTGCCATTGGAGGCAGGGAGATACAAACACAGCCATTTGAAAACCTTGCCAGCCATGAGCACCCCACCACCTGCCTACTGTAA

**ORF5:** ATGAGCACCCCACCACCTGCCTACTGTAATCACACCAGCTTTGAATGCAGGGTGAAAAAGGTGATTGTTCATCCATCCAGAAATCCCACCCAAAAGCATGAATAA

**ORF6:** ATGTGCAGGTGTCTTGAAAGCAGATTAAAAACTCGAGCTGAGGAATTGAGCCTCCTCTTGGAAATGAGCACTGGAGGCAGAGGAATGCTGTAA

**Table S3.** Oligonucleotide sequences in this study.

| **Fragment name** | **Sequences (5’ to 3’)** |
| --- | --- |
| ASO-MYH1G-AS | ACAGCCATTTGAAAACCTTG |
| si-SMAD3 | CACCAGGATGTAACTTGAA |
| si-SP2 | CCATGAACAACCTGGTGAA |
| si-ALKBH5 | GGTGTAAGTTCCAGTTCAA |
| si-FGF18 | GCAAGGAGTGCGTCTTCAT |
| si-SMARCA5 | GCTGCTAACTGGAACTCCA |
| si-SMAD4 | CCTTCGACCTCAAATACGA |
| si-POU2F1 | GTTCATCATCTCACAAACA |

**Table S4.** Differential expression analysis of genes between pectoralis major and soleus in 7-week-old Xinghua chicken

Separate Excel file.

**Table S5.** Differential expression analysis of lncRNAs between pectoralis major and soleus in 7-week-old Xinghua chicken

Separate Excel file.

**Table S6.** Differential analysis of ATAC-seq peaks between pectoralis major and soleus in 7-week-old Xinghua chicken.

Separate Excel file.

**Table S7**. List of open chromatin-associated lncRNAs.

Separate Excel file.

**Table S8**. Differential expression analysis of genes between control group and *MYH1G-AS* interference in CPMs

Separate Excel file.

**Table S9**. Comparative metabolome analysis of control group versus lncRNA *MYH1G-AS* knockdown gastrocnemius.

Separate Excel file.

**Table S10**. lncRNA *MYH1G-AS* specific binding proteins identified by RNA pull-down coupled to mass spectrometry

Separate Excel file.

**Table S11**. FGF18 specific interacting proteins identified by Co-IP coupled to mass spectrometry

Separate Excel file.


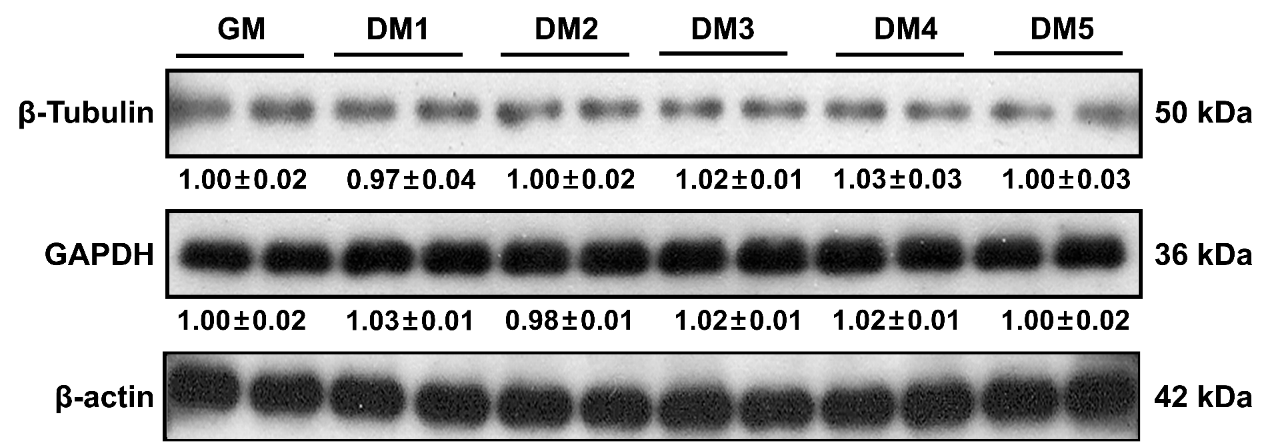


**Fig. S1 Relative β-Tubulin and GAPDH protein expression in myoblast proliferation (myoblast cultured in growth medium [GM]) and differentiation (myoblast cultured in differentiation medium from 1 to 5 day [DM1 to DM5; DM indicate differentiation day]) periods.** The numbers shown below the bands were folds of band intensities relative to control. Band intensities were quantified by ImageJ and normalized to β-Tubulin. Data are expressed as a fold-change relative to the control. Results are presented as mean ± SEM.


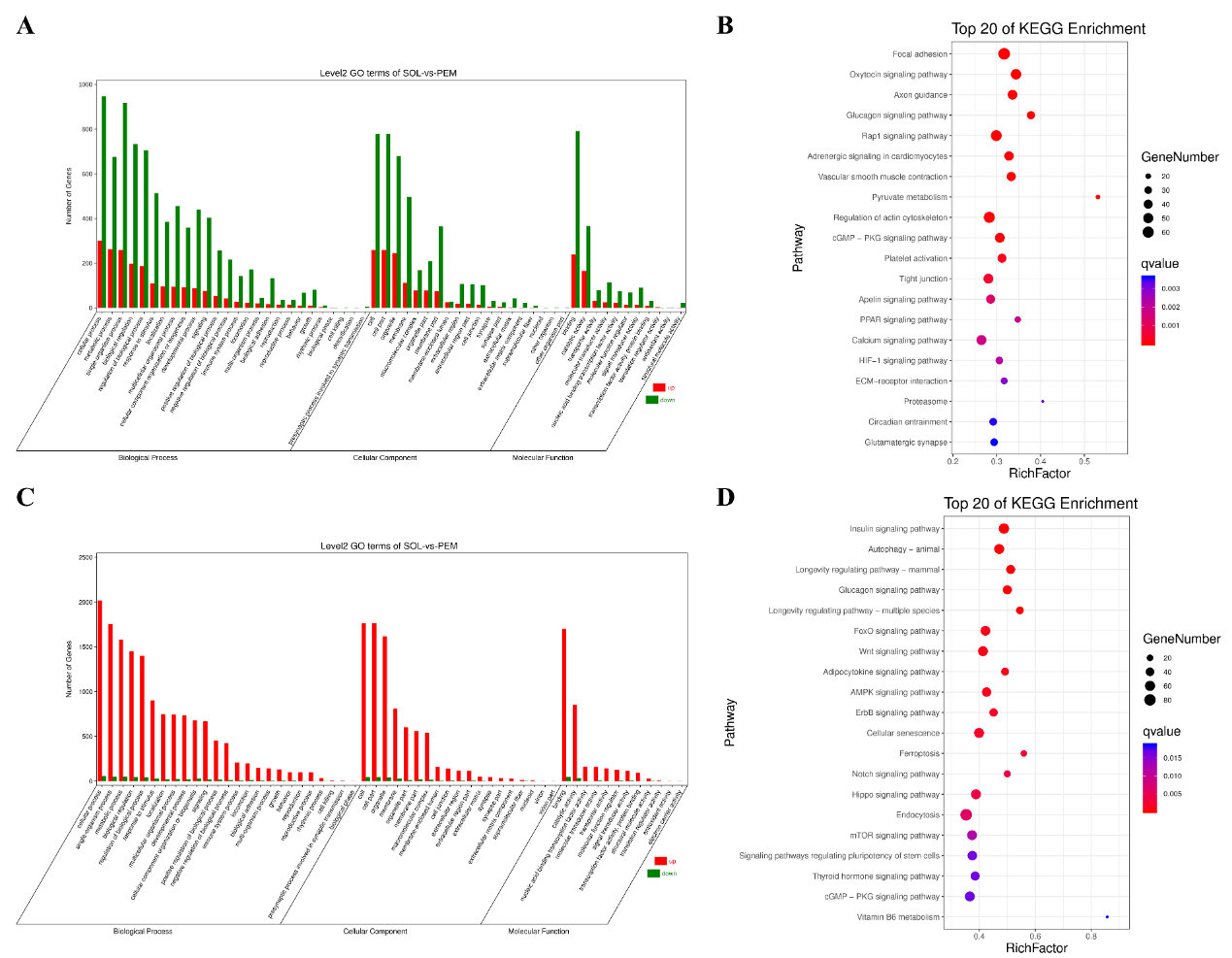


**Fig. S2 GO functions and KEGG pathways analysis of differentially expressed genes and ATAC-seq peaks between pectoralis major and soleus in 7-week-old Xinghua chicken.** (**A**-**B**) GO functions (**A**) and KEGG pathways (**B**) analysis of differentially expressed genes between pectoralis major (PEM) and soleus (SOL) in 7-week-old Xinghua chicken. (**C**-**D**) GO functions (**C**) and KEGG pathways (**D**) analysis of differentially expressed ATAC-seq peaks between PEM and SOL in 7-week-old Xinghua chicken.

**
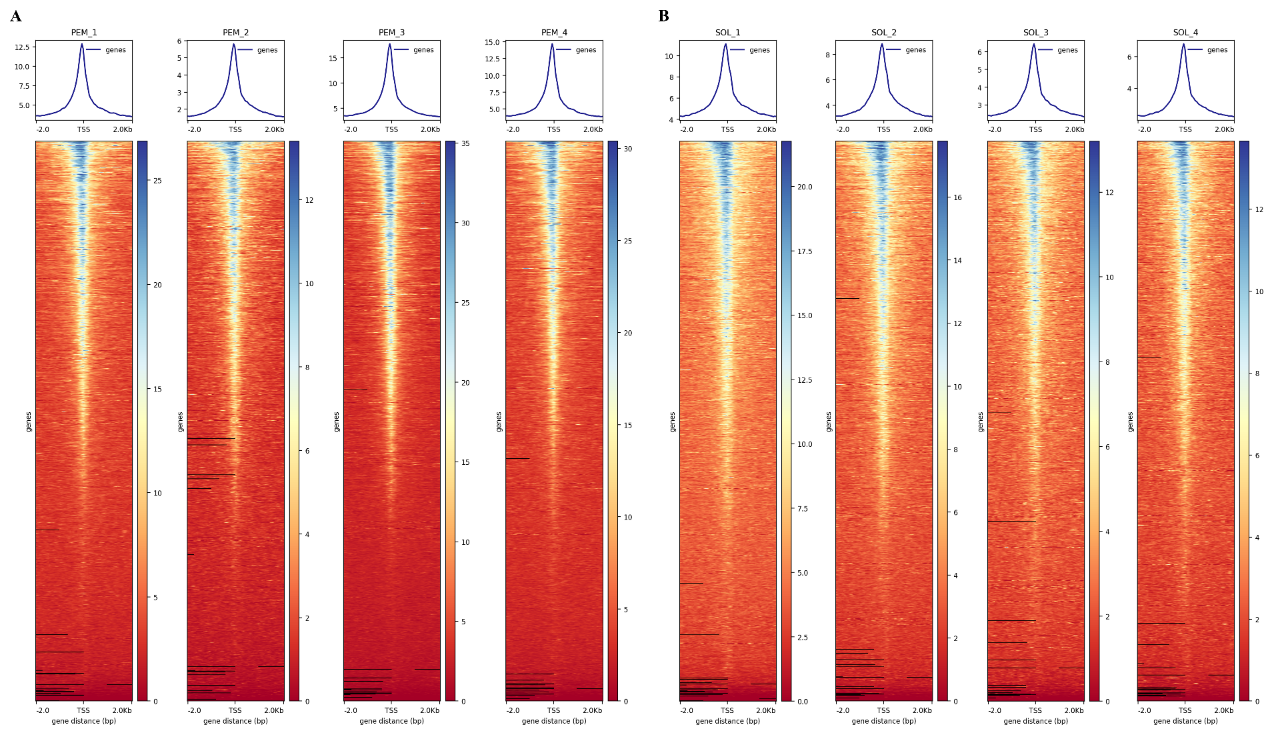
**

**Fig. S3 Heatmap of ATAC-seq signals at transcriptional start site.** (**A**) Heatmap of ATAC-seq signals at transcriptional start site (TSS) in PEM samples. (**B**) Heatmap of ATAC-seq signals at TSS in SOL samples.

**
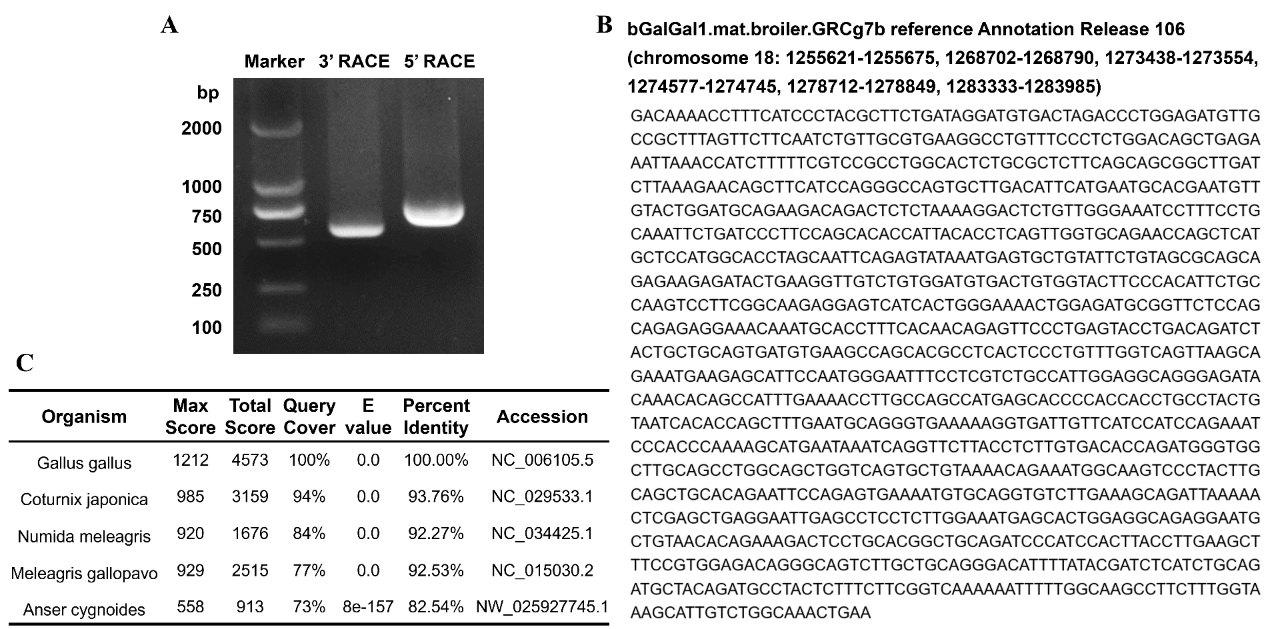
**

**Fig. S4 Characterization of *MYH1G-AS*.** (**A**) Results of *MYH1G-AS* 3’ RACE and 5’ RACE. (**B**) The full-length sequence of *MYH1G-AS*. Coordinates are listed according to bGalGal1.mat.broiler.GRCg7b reference Annotation Release 106. (**C**) Conservative analysis of *MYH1G-AS* performed by using the NCBI’s BLAST. A total of eighteen species, including *Anas platyrhynchos*, *Anser cygnoides*, *Apteryx mantelli mantelli*, *Aquila chrysaetos*, *Bos taurus*, *Coturnix japonica*, *Gallus gallus*, *Geospiza fortis*, *Homo sapiens*, *Meleagris gallopavo*, *Melopsittacus undulatus*, *Mus musculus*, *Numida meleagris*, *Ovis aries*, *Pan troglodytes*, *Rattus norvegicus*, *Sus scrofa* and *Zebra finch* were used for Nucleotide BLAST. Top 5 most conservative results were listed above.

**
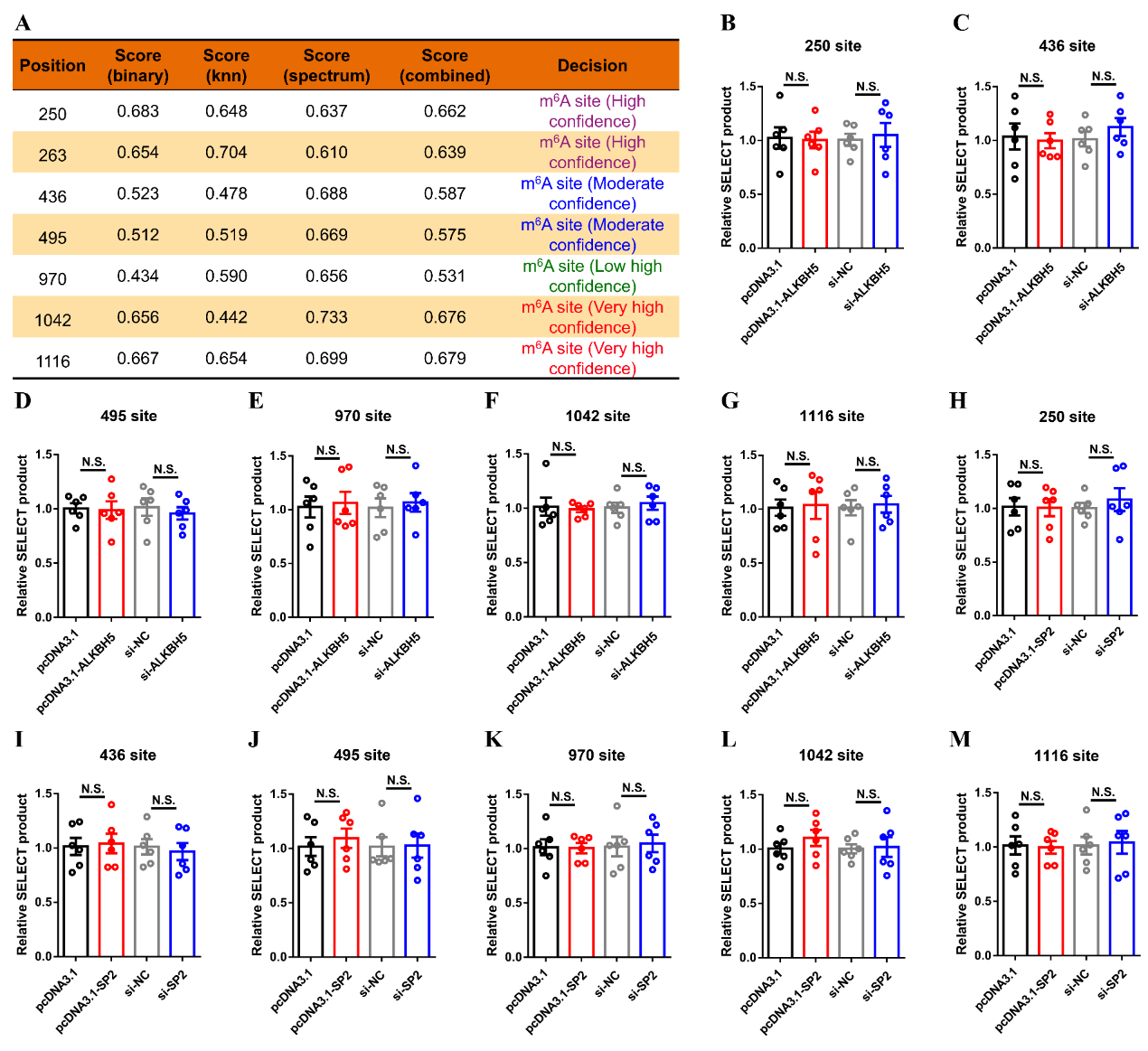
**

**Fig. S5 Prediction and identification of potential m^6^A modification sites on *MYH1G-AS*.** (**A**) Potential m^6^A modification sites on *MYH1G-AS* were predict by using the SRAMP (http://www.cuilab.cn/sramp) software. (**B**-**G**) Relative single-base elongation and ligation-based PCR amplification method (SELECT) product at 250 (**B**), 436 (**C**), 495 (**D**), 970 (**E**), 1042 (**F**), and 1116 (**G**) sites of *MYH1G-AS* after *ALKBH5* overexpression or interference. (**H**-**M**) Relative SELECT product at 250 (**H**), 436 (**I**), 495 (**J**), 970 (**K**), 1042 (**L**), and 1116 (**M**) sites of *MYH1G-AS* after *ALKBH5* overexpression or interference. Results are presented as mean ± SEM. In panels (**B**-**M**), the statistical significance of differences between means was assessed using an independent sample *t*-test. (N.S., no significant difference).

**
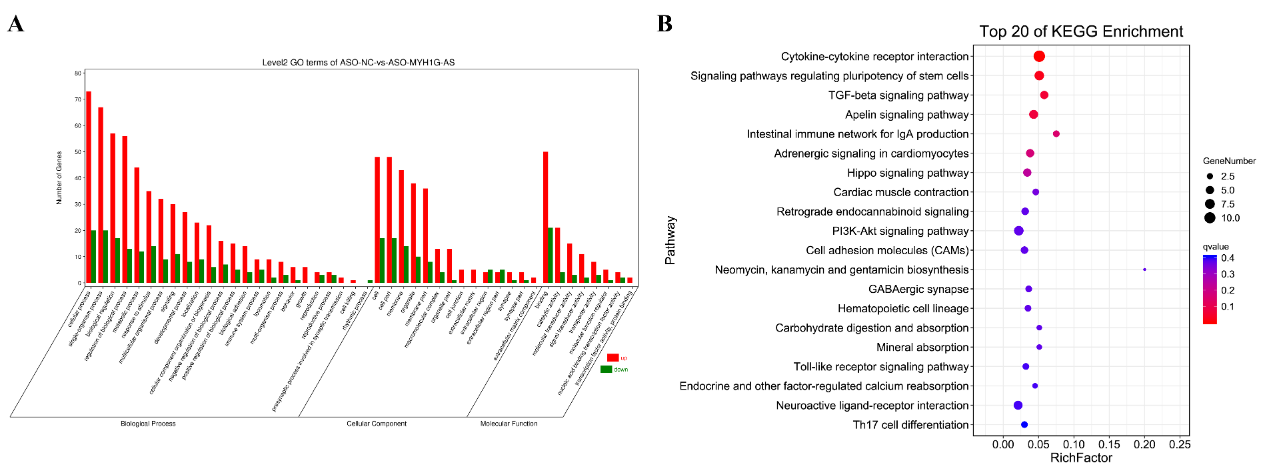
**

**Fig. S6 GO functions and KEGG pathways analysis of** **differentially expressed genes between control group and *MYH1G-AS* interference.** (**A**) GO functions analysis of differentially expressed genes between control group and *MYH1G-AS* interference. (**B**) KEGG pathways analysis of differentially expressed genes between control group and *MYH1G-AS* interference.


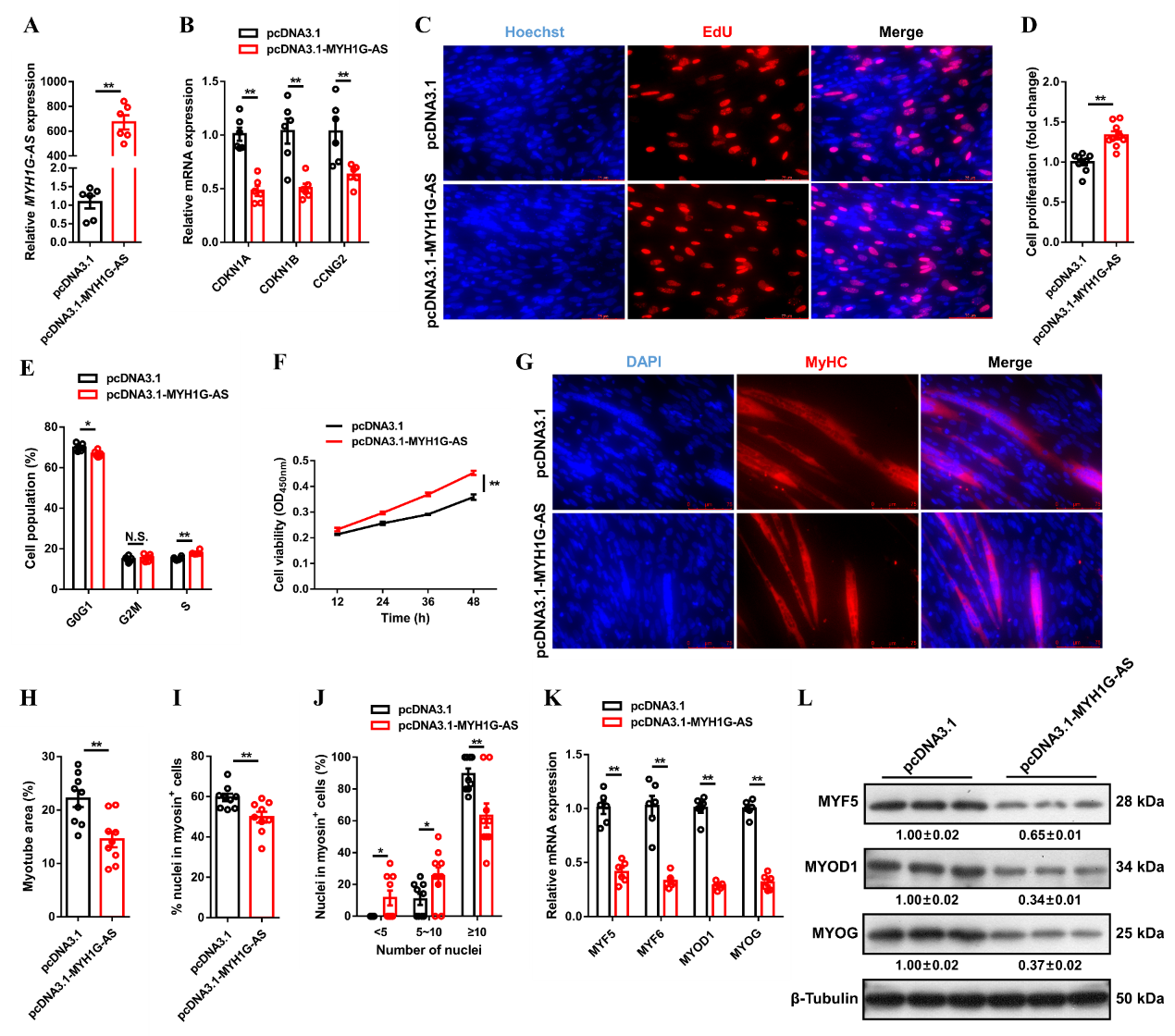


**Fig. S7 Overexpression of *MYH1G-AS* promotes myoblast proliferation but inhibits myoblast differentiation**. (**A**-**L**) Relative *MYH1G-AS* expression (**A**), relative mRNA expressions of several cell cycle-inhibiting genes (**B**), EdU proliferation assays (**C**), proliferation rate of myoblasts (**D**), cell cycle analysis €, CCK-8 assays (**F**), MyHC immunostaining (**G**), myotube area (**H**), differentiation index (**I**), myoblast fusion index (**J**), and relative mRNA (**K**) and protein (**L**) expression levels of myoblast differentiation marker genes with *MYH1G-AS* overexpression *in vitro*. In panel (**L**), the numbers shown below the bands were folds of band intensities relative to control. Band intensities were quantified by ImageJ and normalized to β-Tubulin. Data are expressed as a fold-change relative to the control. Results are presented as mean ± SEM. In panels (**A**-**B**, **D**-**F**, and **H**-**K**), statistical significance of differences between means was assessed using independent sample *t*-test. (* *P* < 0.05; ** *P* < 0.01).


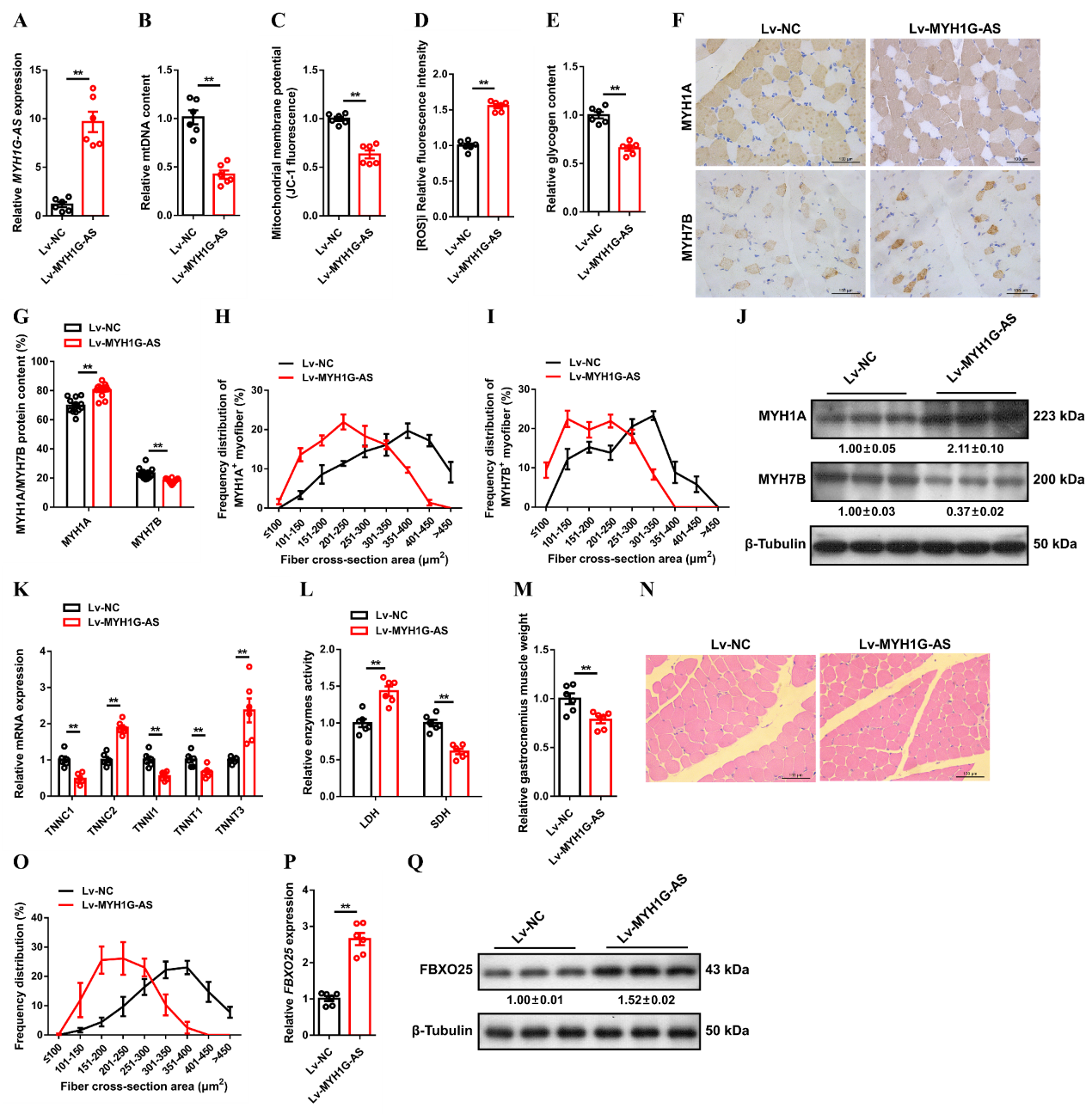


**Fig. S8 Overexpression of *MYH1G-AS* represses mitochondria biogenesis to drive the transformation of slow-twitch to fast-twitch myofiber and induces muscle atrophy**. (**A**-**Q**) Relative *MYH1G-AS* expression (**A**), relative mtDNA content (**B**), mitochondrial membrane potential (**C**), intracellular ROS ([ROS]i) (**D**), relative glycogen content (**E**), immunohistochemistry analysis of MYH1A/MYH7B (**F**), MYH1A/MYH7B protein content (**G**), frequency distribution of MYH1A^+^ (**H**) and MYH7B^+^ (**I**) myofiber CSA, relative protein expression of MYH1A and MYH7B (**J**), relative mRNA expression of several fast- and slow-twitch myofiber genes (**K**), relative enzymes activity of LDH and SDH (**L**), relative gastrocnemius muscle weight (**M**), H&E staining (**N**), frequency distribution of fiber CSA (**O**), and relative mRNA (**P**) and protein (**Q**) expression of *FBXO25* in gastrocnemius with *MYH1G-AS* overexpression. In panel (**J** and **Q**), the numbers shown below the bands were folds of band intensities relative to control. Band intensities were quantified by ImageJ and normalized to β-Tubulin. Data are expressed as a fold-change relative to the control. Results are showed as mean ± SEM. In panels (**A**-**E**, **G**, **K**-**M**, and **P**), statistical significance of differences between means was assessed using independent sample *t*-test. (* *P* < 0.05; ** *P* < 0.01).

**
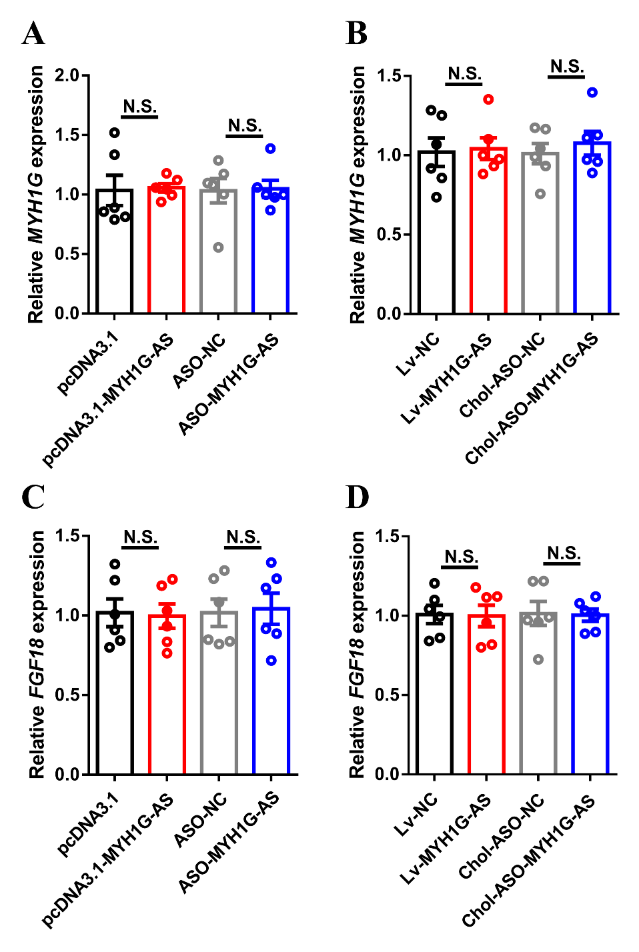
**

**Fig. S9** **The mRNA level of *MYH1G* and *FGF18* didn’t change with *MYH1G-AS* overexpression and knockdown both *in vitro* and *in vivo*.** (**A**-**B**) Relative mRNA expression of *MYH1G* with *MYH1G-AS* overexpression and knockdown *in vitro* (**A**) and *in vivo* (**B**). (**C**-**D**) Relative mRNA expression of *FGF18* with *MYH1G-AS* overexpression and knockdown *in vitro* (**C**) and *in vivo* (**D**). Results are shown as mean ± SEM. In all panels, statistical significance of differences between means was assessed using independent sample *t*-test. (N.S., no significant difference).

**
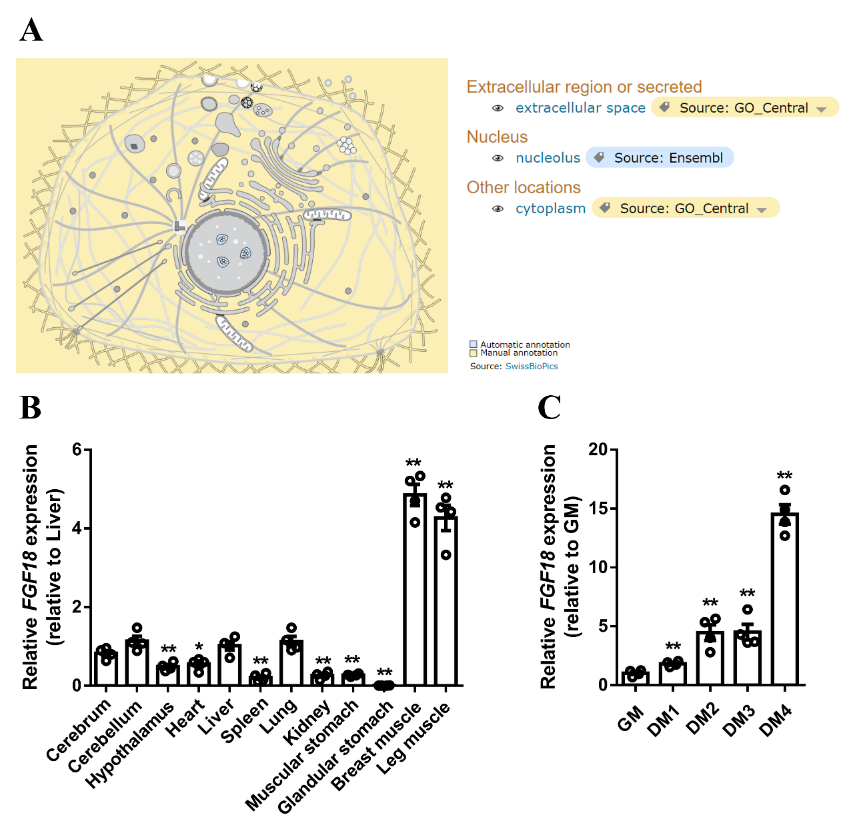
**

**Fig. S10** **The location and expression analysis of *FGF18*.** (**A**) Subcellular location of FGF18 protein annotated by UniProt Knowledgebase (<https://www.uniprot.org/>). (**B**) Tissue expression proﬁles of *FGF18*. The horizontal axis and vertical axis indicate different tissues and their relative expression values, respectively. (**C**) Relative *FGF18* expression during CPM proliferation and differentiation. Results are presented as mean ± SEM. In panels (**B**-**C**), statistical significance of differences between means was assessed using independent sample *t*-test. (* *P* < 0.05; ** *P* < 0.01).


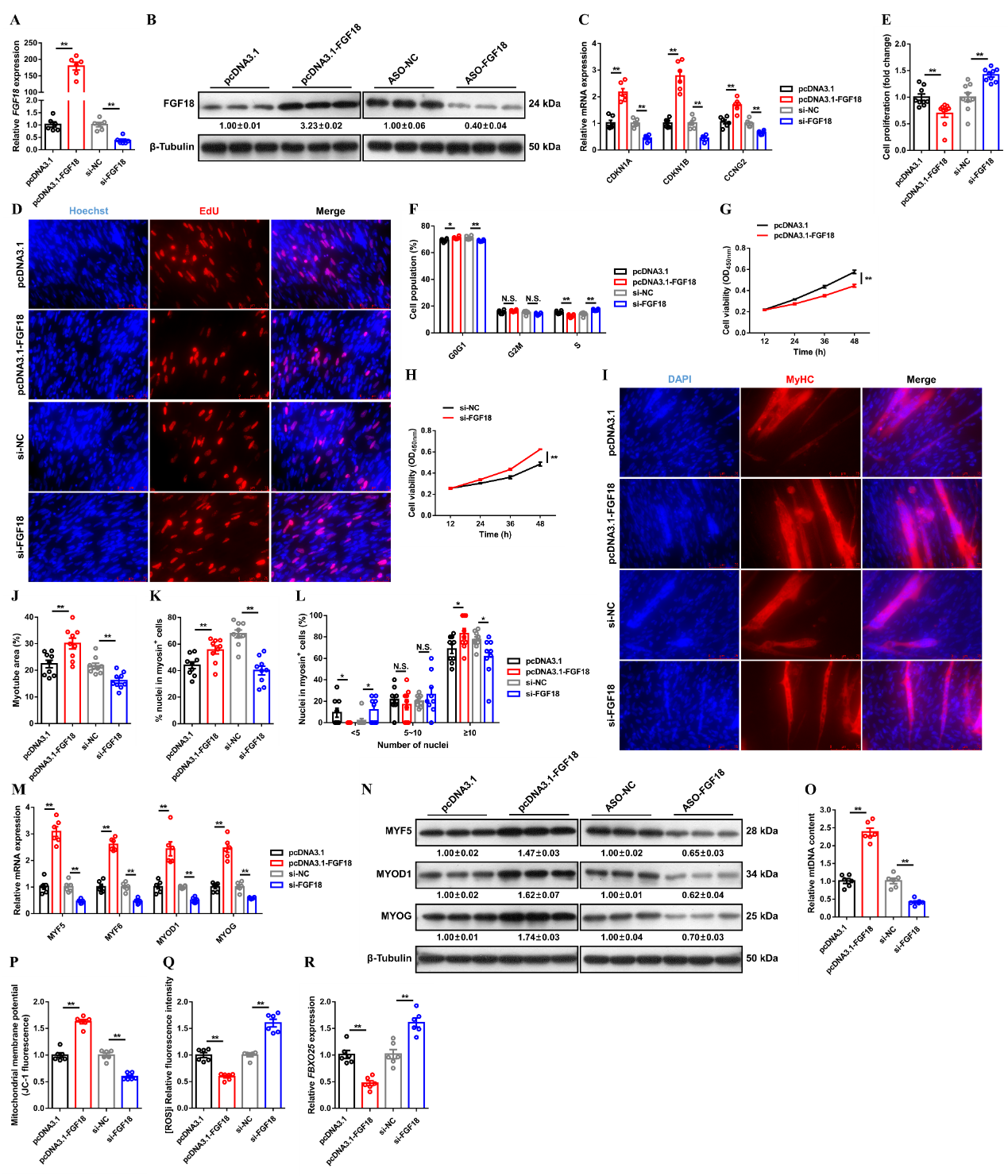


**Fig. S11 *FGF18* inhibits myoblast proliferation, promotes myoblast differentiation, and facilitates mitochondria biogenesis.** (**A**-**R**) Relative mRNA (**A**) and protein **(B**) expression levels of *FGF18*, relative mRNA expressions of several cell cycle-inhibiting genes (**C**), EdU proliferation assays (**D**), proliferation rate of myoblasts (**E**), cell cycle analysis (**F**), CCK-8 assays (**G**-**H**), MyHC immunostaining (**I**), myotube area (**J**), differentiation index (**K**), myoblast fusion index (**L**), relative mRNA (**M**) and protein (**N**) expression levels of myoblast differentiation marker genes, relative mtDNA content (**O**), mitochondrial membrane potential (**P**), intracellular ROS ([ROS]i) (**Q**), and relative *FBXO25* expression (**R**) with *FGF18* overexpression or interference *in vitro*. In panels (**B** and **N**), the numbers shown below the bands were folds of band intensities relative to control. Band intensities were quantified by ImageJ and normalized to β-Tubulin. Data are expressed as a fold-change relative to the control. Results are showed as mean ± SEM. In panels (**A**, **C**, **E**-**H**, **J**-**M**, and **O**-**R**), statistical significance of differences between means was assessed using independent sample *t*-test. (* *P* < 0.05; ** *P* < 0.01).


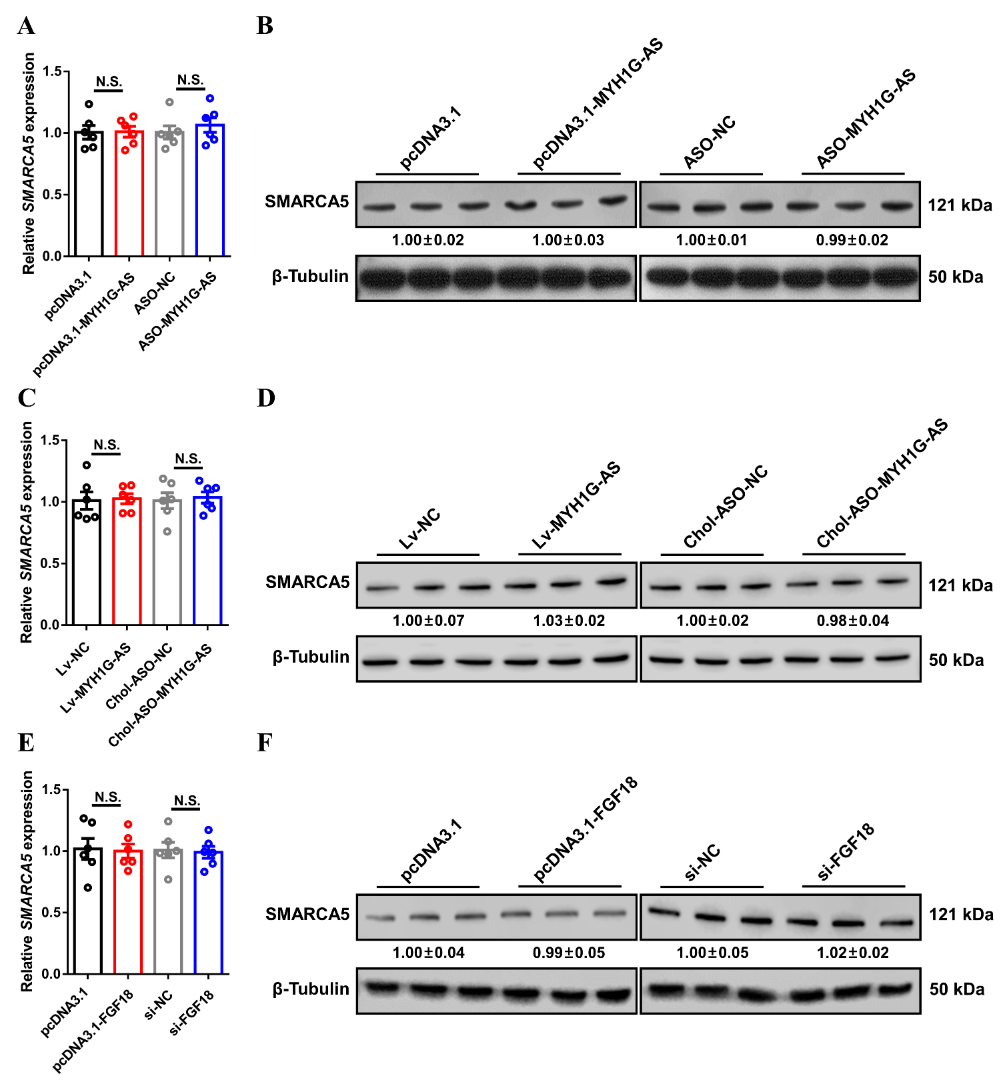


**Fig. S12** **Neither *MYH1G-AS* nor *FGF18* regulate the mRNA and protein expression of *SMARCA5*.** (**A**-**D**) Relative mRNA (**A** and **C**) and protein (**B** and **D**) expression levels of *SMARCA5* after *MYH1G-AS* overexpression or interference *in vitro* (**A**-**B**) or *in vivo* (**C**-**D**). (**E**-**F**) Relative mRNA (**E**) and protein (**F**) expression levels of *SMARCA5* after *FGF18* overexpression or interference. In panels (**B**, **D**, and **F**), the numbers shown below the bands were folds of band intensities relative to control. Band intensities were quantified by ImageJ and normalized to β-Tubulin. Data are expressed as a fold-change relative to the control. Results are presented as mean ± SEM. In panels (**A**, **C**, and **E**), statistical significance of differences between means was assessed using independent sample *t*-test. (N.S., no significant difference).


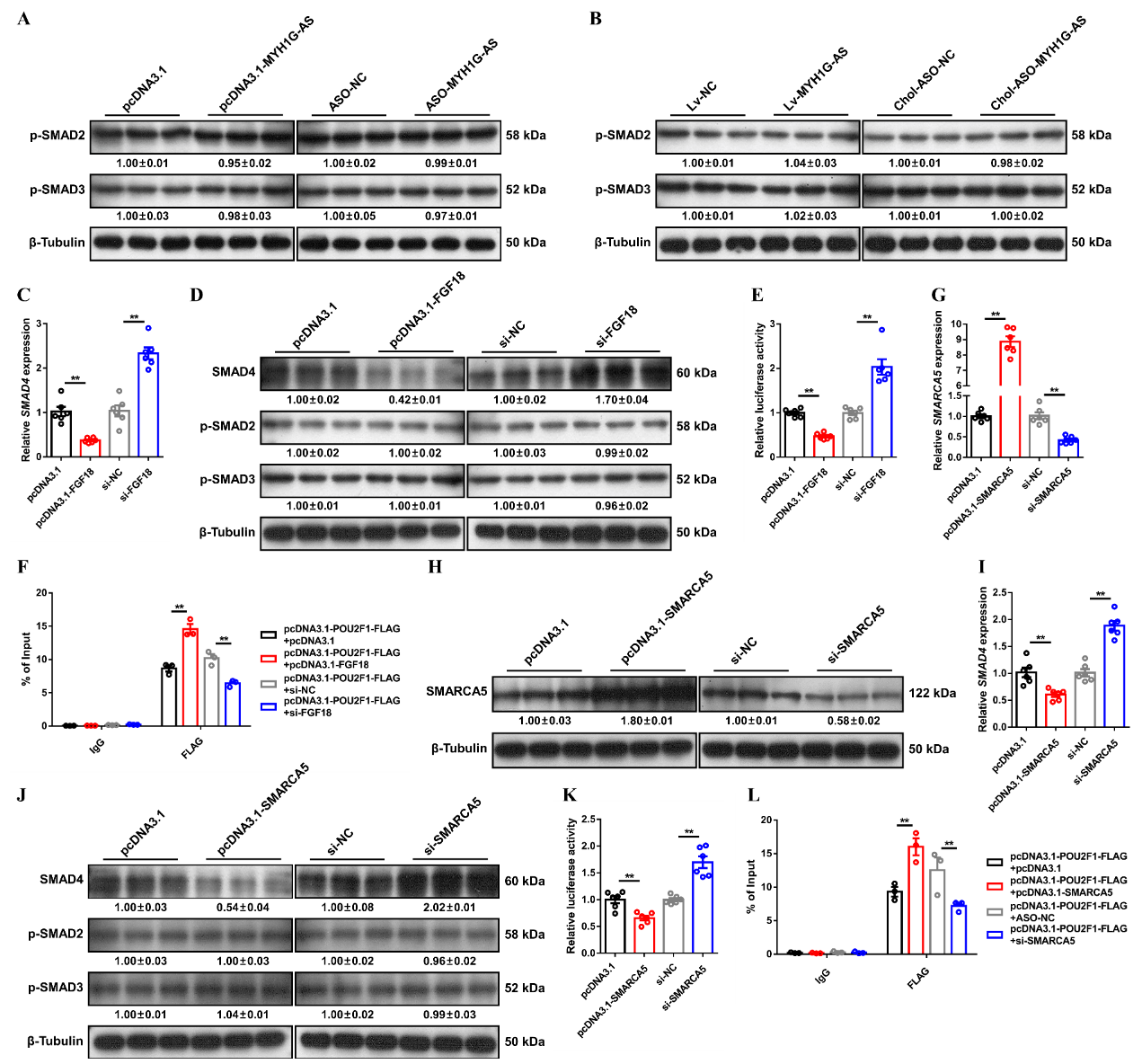


**Fig. S13 *FGF18* and *SMARCA5* promotes the expression and transcription of *SMAD4*.** (**A**-**B**) Protein expression levels of phosphorylated SMAD2 and phosphorylated SMAD3 after *MYH1G-AS* overexpression or interference *in vitro* (**A**) or *in vivo* (**B**). (**C**-**F**) Relative *SMAD4* mRNA expression (**C**), protein expression levels of *SMAD4*, phosphorylated SMAD2 and phosphorylated SMAD3 (**D**), the transcriptional activity of *SMAD4* core promoter region (**E**), and ChIP analysis of the binding capacity of POU2F1 to *SMAD4* promoter (**F**) with *FGF18* overexpression or knockdown. (**G**-**L**) Relative mRNA (**G**) and protein (**H**) expression levels of *SMARCA5*, relative *SMAD4* mRNA expression (**I**), protein expression levels of *SMAD4*, phosphorylated SMAD2 and phosphorylated SMAD3 (**J**), the transcriptional activity of *SMAD4* core promoter region (**K**), and ChIP analysis of the binding capacity of POU2F1 to *SMAD4* promoter (**L**) with *SMARCA5* overexpression or knockdown. In panels (**A**-**B**, **D**, **H**, and **J**), the numbers shown below the bands were folds of band intensities relative to control. Band intensities were quantified by ImageJ and normalized to β-Tubulin. Data are expressed as a fold-change relative to the control. Results are presented as mean ± SEM. In panels (**C**, **E**-**G**, **I**, and **K**-**L**), statistical significance of differences between means was assessed using independent sample *t*-test. (* *P* < 0.05; ** *P* < 0.01).
